# Pre-clinical evaluation of the efficacy and safety of human induced pluripotent stem cell-derived cardiomyocyte patch

**DOI:** 10.1186/s13287-024-03690-8

**Published:** 2024-03-13

**Authors:** Shigeru Miyagawa, Takuji Kawamura, Emiko Ito, Maki Takeda, Hiroko Iseoka, Junya Yokoyama, Akima Harada, Noriko Mochizuki-Oda, Yukiko Imanishi-Ochi, Junjun Li, Masao Sasai, Fumiyo Kitaoka, Masaki Nomura, Naoki Amano, Tomoko Takahashi, Hiromi Dohi, Eiichi Morii, Yoshiki Sawa

**Affiliations:** 1 Department of Cardiovascular Surgery, Graduate School of Medicine, Osaka University, Suita, Osaka 565-0871 Japan; 2 Center for iPS Cell Research and Application (CiRA), Kyoto University, Kyoto, 606-8507 Japan; 3 Department of Environmental Preventive Medicine (Yamada Bee Company, Inc.), Center for Preventive Medical Sciences, Chiba University, Chiba, 263-8522 Japan; 4 Department of Histopathology, Graduate School of Medicine, Osaka University, Suita, Osaka 565-0871 Japan

**Keywords:** Stem cell therapy, Ischemic heart failure, Myocardial infarction, Cardiomyocyte patch, Regenerative therapy

## Abstract

**Background:**

Cell- or tissue-based regenerative therapy is an attractive approach to treat heart failure. A tissue patch that can safely and effectively repair damaged heart muscle would greatly improve outcomes for patients with heart failure. In this study, we conducted a preclinical proof-of-concept analysis of the efficacy and safety of clinical-grade human induced pluripotent stem cell-derived cardiomyocyte (hiPSC-CM) patches.

**Methods:**

A clinical-grade hiPSC line was established using peripheral blood mononuclear cells from a healthy volunteer that was homozygous for human leukocyte antigens. The hiPSCs were differentiated into cardiomyocytes. The obtained hiPSC-CMs were cultured on temperature-responsive culture dishes for patch fabrication. The cellular characteristics, safety, and efficacy of hiPSCs, hiPSC-CMs, and hiPSC-CM patches were analyzed.

**Results:**

The hiPSC-CMs expressed cardiomyocyte-specific genes and proteins, and electrophysiological analyses revealed that hiPSC-CMs exhibit similar properties to human primary myocardial cells. In vitro and in vivo safety studies indicated that tumorigenic cells were absent. Moreover, whole-genome and exome sequencing revealed no genomic mutations. General toxicity tests also showed no adverse events posttransplantation. A porcine model of myocardial infarction demonstrated significantly improved cardiac function and angiogenesis in response to cytokine secretion from hiPSC-CM patches. No lethal arrhythmias were observed.

**Conclusions:**

hiPSC-CM patches are promising for future translational research and may have clinical application potential for the treatment of heart failure.

**Supplementary Information:**

The online version contains supplementary material available at 10.1186/s13287-024-03690-8.

## Background

Heart failure remains correlated with a high mortality rate despite advances in medical treatment. Novel treatment techniques are necessary to improve the outcomes of heart failure patients. Recent studies have illustrated that human induced pluripotent stem cells (hiPSCs) are a source of stem cells that can replace lost cells in diseased organs [1–3]. Moreover, hiPSC-derived cardiomyocytes (hiPSC-CMs), in the form of myocardial tissue patches, can be used to supply new cardiomyocytes to the heart, suggesting their potential clinical application in the treatment of heart failure [4–7]. However, there are major concerns regarding the safety and, particularly, the tumorigenicity of hiPSCs [8–10]. Adequate in vitro and in vivo preclinical studies on the safety and efficacy of hiPSC-CMs are necessary for the initiation of subsequent clinical trial investigations and the potential clinical application of this technique.

In this study, we determined whether a clinical-grade hiPSC-CM patch could serve as a functional myocardial tissue. We performed a preclinical study to ensure its safety and conducted a proof-of-concept analysis of hiPSC-CM patches in clinical applications.

## Methods

### Study design

We analyzed the cellular characteristics, efficacy, and safety of clinical-grade hiPSCs, hiPSC-CMs, and hiPSC-CM patches. To analyze the cellular characteristics, we assessed gene and protein expression, electrical propagation, contractile force, contractility, and calcium levels in vitro. To determine the safety of hiPSC-CM patches, we evaluated the presence of tumorigenicity-related cells using qRT‒PCR, cell growth assays, soft agar colony formation assays, whole-genome/exome sequencing analysis, and SNP array analysis in vitro and in NOD/Shi-scid, IL-2R γ^null^ (NOG) mice in vivo. Furthermore, to assess the efficacy of hiPSC-CM patches, we measured cardiac function, fibrosis, angiogenesis, and arrhythmogenesis in a porcine myocardial infarction (MI) model. Experimental methods are described in the Supplementary Materials. Immunofluorescence was performed using the primary and secondary antibodies listed in Additional file 1: Table S1, and qRT‒PCR primer sequences are listed in Additional file 1: Table S2.

## Clinical-grade hiPSCs

The clinical-grade-hiPSC line QHJI14s04 was established from peripheral blood mononuclear cells collected from a healthy HLA homozygous (HLA-A, HLA-B, HLA-C, HLA-DRB1, HLA-DPB1, and HLA-DQB1) donor that possessed the most frequent haplotype in the Japanese population [11]. The QHJI14s04 cell line was generated using episomal plasmids (pCE-hSK, pCE-hUL, pCE-hOCT3/4, pCE-mp53DD, and pCXB-EBNA1; Additional file 1: Table S3) [12] and maintained using a feeder-free and xeno-free culture system [13] in the cell processing center of the Center for iPS Cell Research and Application (CiRA; Kyoto University, Kyoto, Japan). We performed tests to ensure sterility and identity, as well as characterization tests, including the evaluation of cell morphology, viability, vector retention, stem cell marker expression, and genomic analysis (Additional file 1: Table S4).

### Generation of the master cell bank (MCB)

We received two cryovials of hiPSC passage (P) 10 from CiRA. One vial was used for the growth rate evaluation and culture condition optimization, whereas the other cryovial was used to produce the MCB. The hiPSC (P10) cells were expanded up to P12 and cryopreserved at P12 to produce the MCB using qualified reagents and materials from the Center for Gene and Cell Processing of Takara Bio Inc. (Kusatsu, Japan), which complies with good manufacturing practices (GMP)/good gene, cellular, and tissue-based product manufacturing practice standards. Cells and supernatants used for analysis were prepared by subculturing P12 cells for two additional passages. In accordance with ICH Q5A and 5D, the MCB was inspected using DNA fingerprinting and electron microscopy and evaluated for sterility, reverse transcriptase activity, and presence of mycoplasma, human viruses, or infectious retroviruses (in vitro and in vivo) (Additional file 1: Table S5).

### hiPSC-CM patch preparation

Prior to cell seeding, temperature-responsive cell culture dishes (UpCell; CellSeed, Tokyo, Japan) were filled with Dulbecco’s Modified Eagle Medium (DMEM) (Nacalai Tesque, Kyoto, Japan) supplemented with 20% fetal bovine serum (FBS) (Sigma‒Aldrich, St. Louis, MO, USA) and incubated overnight. After freeze‒thawing, the hiPSC-CMs were plated in the UpCell-coasted dishes at a density of 2.3 × 10^6^ cells/cm^2^ in DMEM containing 20% FBS and cultured at 37 ℃ and 5% CO_2_. The medium was changed on the following day and 2 d after cell seeding. After culturing for 72 h, the hiPSC-CM patches were harvested and washed gently with Hank’s balanced salt solution (Thermo Fisher Scientific, Waltham, MA, USA).

### Statistical analysis

Statistical significance of in the in vitro experiments was determined via a two-tailed Student’s *t* test. JMP Pro 13 software (SAS Institute Inc., Cary, NC) was used for statistical analysis of the in vivo porcine model efficacy experiment. Continuous data are expressed as the mean ± SD. The analyses were performed using nonparametric methods since the sample sizes were too small to determine whether the distribution was normal or skewed. Within-group differences were compared using the Wilcoxon signed-rank test; between-group differences were compared with the Wilcoxon–Mann–Whitney *U* test. *P* values < 0.05 were considered statistically significant.

### Study approval

This study complied with the Declaration of Helsinki. The ethics committees approved all research protocols and informed consent was obtained from each participant (Approval number 14306-7). All experimental procedures and protocols involving animals were performed in accordance with the national regulations and the Animal Research: Reporting of In Vivo Experiments (ARRIVE) guidelines, reviewed by the Committee for Animal Experiments, and approved by the president of Osaka University (Approval number 25–110-012 and 30–019-010).

## Results

### Establishing the master cell bank

The human iPS cell line QHJI14s04 was established as a clinical-grade hiPSC source by transfecting multiple genes into peripheral blood mononuclear cells collected from a healthy volunteer homozygous for HLA (Additional file 1: Table S3). While establishing hiPSCs and the MCB, quality checks were performed at each point (Additional file 1: Figure S1). The quality of hiPSCs was assessed by determining colony morphology, evaluating the residual plasmid vector used for iPS production, assessing karyotype, confirming the absence of pluripotent markers such as *POU5F1*, *NANOG*, *TRA-1–60*, *TRA-2–49*, and *SSEA-4*, assessing sterility by confirming the absence of mycoplasma, endotoxins, and viruses and performing HLA typing and short tandem repeat (STR) genotyping (Additional file 1: Table S4). We generated MCBs using culture-expanded hiPSCs produced under GMP conditions. The quality of the MCB was confirmed, as there was no contamination by foreign pollutants such as bacteria, mycoplasma, or viruses. Moreover, the MCB was further analyzed via STR genotyping (Additional file 1: Table S5).

### Characterization of hiPSC-CMs and hiPSC-CM patches

Quality checks were performed at each point in the process of producing hiPSC-CMs and hiPSC-CM patches (Additional file 1: Figure S1). To characterize hiPSC-CMs, experiments were performed using thawed cryopreserved hiPSC-CMs that underwent cardiomyogenic differentiation, purification, and elimination of residual undifferentiated hiPSCs during quality check 4 (QC4, Additional file 1: Figure S1). The quality of the hiPSC-CMs was confirmed based on cell viability, purity of cardiomyocytes, sterility, and the absence of mycoplasma and endotoxins (Additional file 1: Table S6). During myocardial differentiation, the number of cells increased. The qPCR and immunohistochemistry results demonstrated that the expression of markers for pluripotency, early mesoderm morphogenesis, cardiac progenitor cells, and cardiomyocytes changed over time (Additional file 1: Figure S2). Analysis of undifferentiated stem cell-related genes revealed that the expression levels of these markers were lower in hiPSC-CMs than in hiPSCs (Additional file 1: Figure S3a). Moreover, analysis of cardiomyocyte differentiation-related genes revealed that several genes in hiPSC-CMs showed expression levels similar to those in human adult or fetal heart tissue samples compared to the expression levels of those genes in undifferentiated hiPSCs (Additional file 1: Figure S3b).

**Fig. 1:**
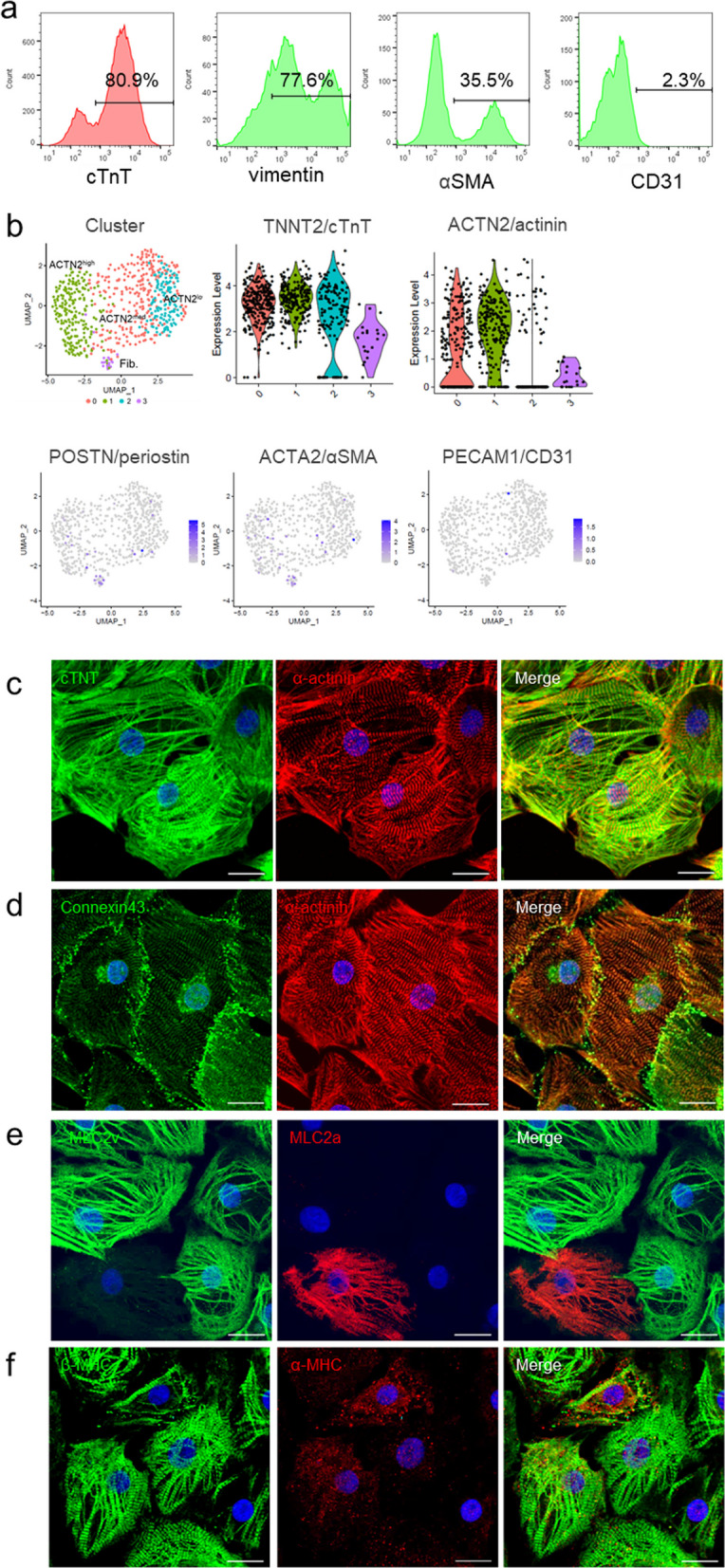
Phenotypic characteristics of cryopreserved hiPSC-CMs after cardiomyogenic differentiation, purification, and elimination of residual undifferentiated hiPSCs. **a**: Populations of cryopreserved hiPSC-CMs were assessed via flow cytometry. The histograms show the expression of vimentin, αSMA, and CD31 in the cTnT-negative cell population (representative data of n = 5). The mean cTnT-positive ratio was 70.3% (n = 5). Scale bar: 1 cm. **b**: Four cell populations in the cryopreserved hiPSC-CMs were determined using single-cell RNA-seq. Violin plots show the expression of the cardiac markers TNNT2 and ACTN2. UMAP plot shows the expression of periostin (a fibroblast marker), SMA (a smooth muscle marker) and CD31 (an endothelial cell marker). **c**–**f**: Structure and morphology of cryopreserved hiPSC-CM. Immunofluorescence of cardiac-specific proteins: **c** cTNT (green) and α-actinin (red), **d** connexin-43 (green) and α-actinin (red), **e** MLC2v (green) and MLC2a (red), and **f** β-MHC (green) and α-MHC (red). Scale bar: 20 μm

The cardiac troponin T (cTNT) marker was present in 60–80% of the hiPSC-CMs. Most cTNT-negative noncardiomyocytes expressed the smooth muscle cell marker αSMA or the fibroblast marker vimentin; however, a few cells (1–5%) expressed the endothelial cell marker CD31 (Fig. 1a). Single-cell RNA-seq identified four cell populations, three of which (clusters 0, 1, and 2) consisted of cardiomyocytes that expressed the cardiac cell marker *TNNT2* and differed in their expression level of *ACTN2*. The fourth population (cluster 3) expressed *POSTN* and *ACTA2*, which are highly expressed in fibroblasts and smooth muscles, respectively (Fig. 1b). In addition, immunohistochemical staining of hiPSC-CMs showed that they express ventricular muscle contractile proteins, such as ventricular isoform of myosin light chain (MLC2v), beta cardiac myosin heavy chain (β-MHC), gap junction protein, and connexin 43 (Fig. 1c). Furthermore, the expression of cardiomyocyte ion channels in hiPSC-CMs was similar to that in adult heart tissue samples (Additional file 1: Figure S3c). The drug response of the hiPSC-CMs was assessed by measuring calcium levels and contractile properties. The administration of isoproterenol resulted in a marked positive inotropic effect, and the administration of proarrhythmic E-4031 resulted in a clear QT prolongation (Additional file 1: Figure S4, S5).

To characterize the hiPSC-CM patches, the following experiments were performed after thawing the cryopreserved hiPSC-CMs and preparing hiPSC-CM patches of a suitable size for each experiment.

**Fig. 2:**
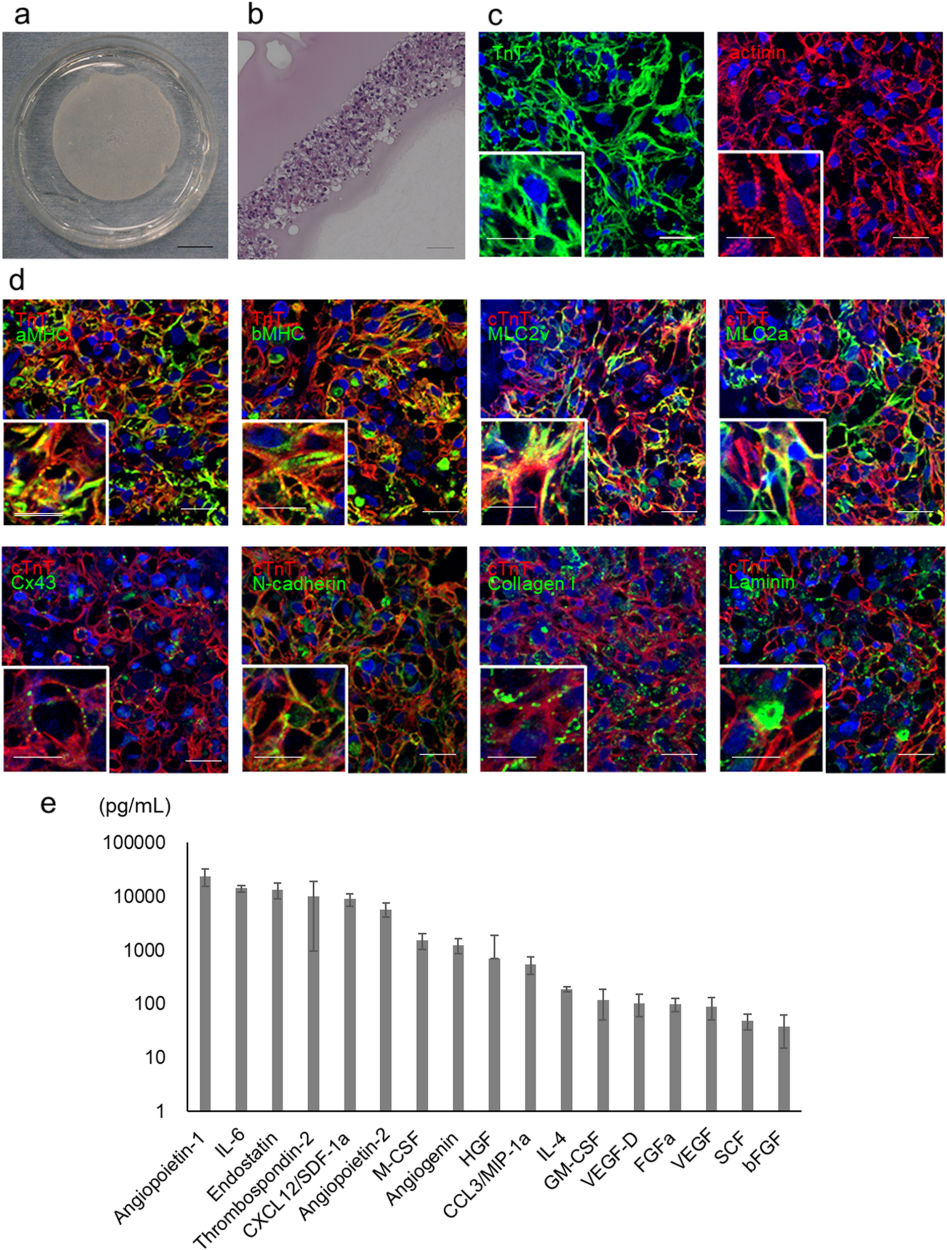

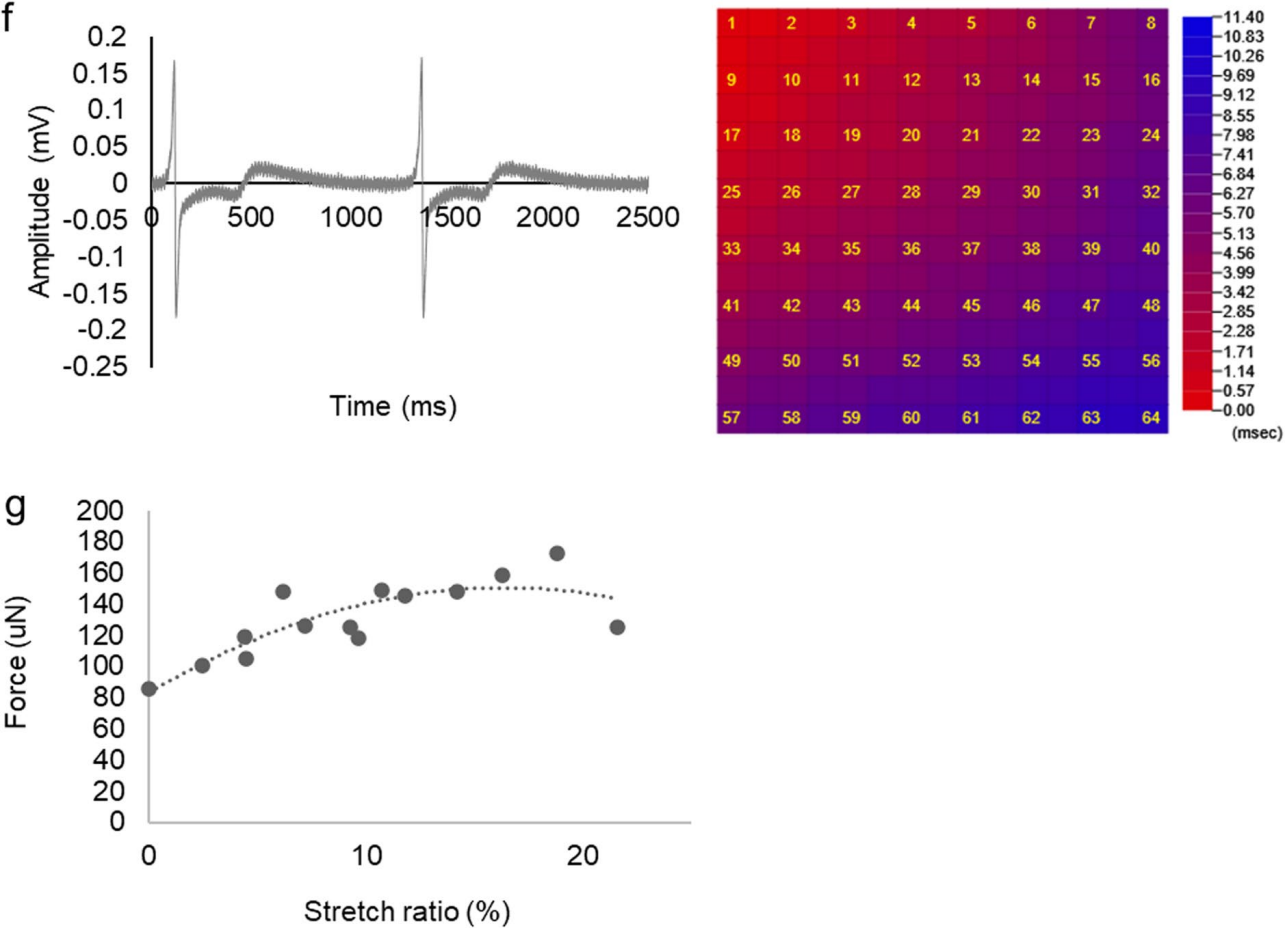
Histological characteristics of the hiPSC-CM patch. **a**: Image of a hiPSC-CM patch in a 6-cm diameter dish. **b**: Hematoxylin and eosin staining of hiPSC-CM patches created on a 24-well plate. Scale bars: 50 μm (**b**). **c**, **d**: Representative image of immunostained hiPSC-CM patches created on a 24-well plate. **c** TnT (green) and actinin (red); **d** TnT (red) and α-MHC, β-MHC, MLC2v, MLC2a, connexin 43 (cx43), N-cadherin, collagen I, and laminin (green). Scale bars: 20 μm, 10 μm (inset image). **e**: In vitro quantification of cytokines and growth factors. The culture supernatant of the hiPSC-CM patches under normoxic conditions (n = 5) was collected and assessed using the Bioplex suspension array system. **f**: Electrophysiological properties of the hiPSC-CM patch created on a 96-well plate. Extracellular field potentials were recorded using a multielectrode array system. A representative extracellular potential waveform and a propagation map are shown. g: The contractile force of the hiPSC-CM patch created on a 96-well plate was assessed using MicroTester G2. The relationship between the contraction force and the stretch rate is shown

Prior to transplantation surgery, hiPSC-CM patches were prepared using temperature-responsive culture dishes (Fig. 2a, b). The hiPSC-CM patches were harvested and cultured for 72 h by lowering the temperature to room temperature (Fig. 2a). Hematoxylin and eosin staining showed that the hiPSC-CMs formed patches in multiple layers (Fig. 2b). To clarify the structural features of hiPSC-CM patches, immunohistochemistry of the following proteins was performed: cardiac structural proteins such as cTNT and α-actinin, cardiac contractile proteins such as alpha-MHC (α-MHC) and β-MHC, atrial isoform of myosin light chain 2 (MLC2a) and MLC2v, cell adhesion- or gap junction-related proteins such as connexin 43 and N-cadherin, and extracellular matrix proteins (ECM) proteins such as collagen I and laminin. The hiPSC-CM patches revealed well-organized sarcomeric structures and upregulated expression of ECM proteins (Fig. 2c, d). Furthermore, the ultrastructure of the hiPSC-CM patch showed myofibrils with transverse Z-bands and a mitochondrial structure (Additional file 1: Figure S6a).

The hiPSC-CM patch also expressed various angiogenic cytokines, such as hepatocyte growth factor (HGF) and vascular endothelial growth factor (VEGF), under normoxic conditions (Fig. 2e). Furthermore, the expression of VEGF and angiogenin was significantly upregulated when the hiPSC-CM patch was cultured under hypoxic conditions, which the patch would be exposed to following transplantation, compared to that under normoxic conditions (Additional file 1: Figure S6b). Furthermore, single-cell RNA-seq of cryopreserved hiPSC-CMs revealed that the gene expression of VEGF was upregulated in clusters 1 and 3 and downregulated in cluster 2 (Additional file 1: Figure S6c). In addition, the hiPSC-CM patch resulted in synchronous, regular, and continuous beating, indicating electrical linkage throughout the patches (Fig. 2f). The hiPSC-CM patch responded in accordance with the Frank–Starling mechanism, where the contraction force of the hiPSC-CM patch increased as its stretch rate increased (Fig. 2g).

### Efficacy study of hiPSC-CM patches in a porcine MI model

**Fig. 3:**
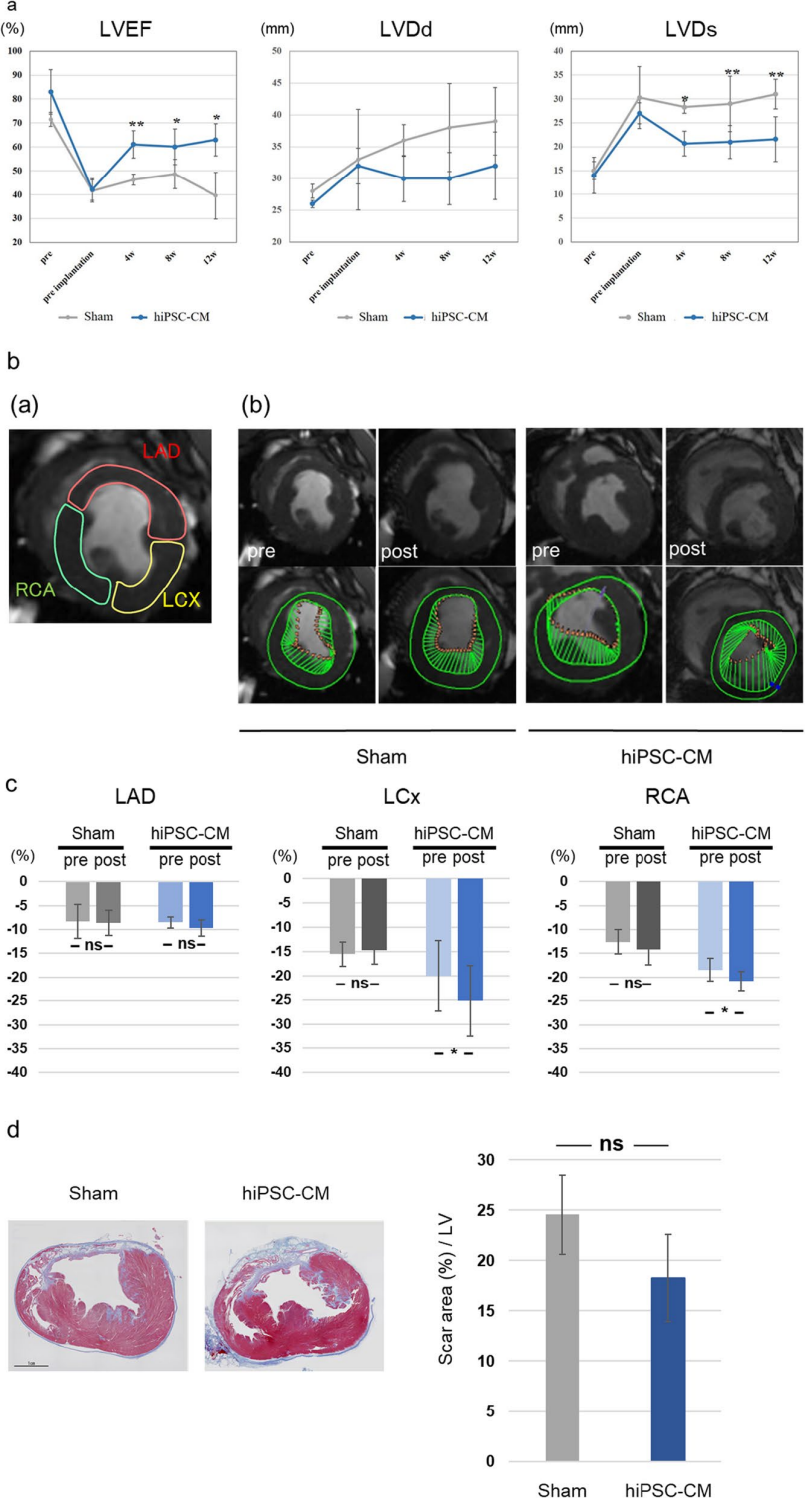
Efficacy of hiPSC-CM patch transplantation in a porcine MI model: **a** preclinical trial. **a**: Change in cardiac function after transplantation of the hiPSC-CM patch in MI model pigs. LVEF, left ventricular ejection fraction; LVDd, left ventricular end-diastolic diameter; LVDs, left ventricular end-systolic diameter. Sham: n = 4, hiPSC-CM: n = 7. **b**: Representative images of endocardial systolic cardiac wall motion at the papillary muscle level 12 weeks after the implantation. (**a**) LAD (red), LCX (yellow), and RCA (green) regions. (**b**) Circumferential strains of sham and hiPSC-CM patches. **c**: Cardiac MRI was performed to compare the LV CS values at baseline and 12 weeks following treatment. Sham: n = 4, hiPSC-CM patch: n = 6. **d**: Pathological interstitial fibrosis 12 weeks following treatment. Left: Masson's trichrome staining of a porcine heart. Scale bar: 1 cm. Right: % area of fibrosis. Data are presented as the mean ± SD. sham: n = 4, hiPSC-CM patch: n = 6. *P < 0.05, **P < 0.01; ns, not significant.

The efficacy of hiPSC-CM patches was evaluated using a porcine infarction model (Additional file 1: Figure S7). Transthoracic echocardiography was performed prior to and 4, 8, and 12 weeks following hiPSC-CM patch transplantation or sham surgery. Before left anterior descending artery (LAD) ligation and before transplantation, no significant differences were observed in the left ventricle (LV) ejection fraction (LVEF), LV end-diastolic diameter (LVDd), and LV end-systolic diameter (LVDs) values between the sham group and the hiPSC-CM group (Fig. 3a). LVEF was significantly greater in the hiPSC-CM patch group than in the sham group after 4 weeks (61.1 ± 5.7% vs. 46.3 ± 2.3%, *P* < 0.01), 8 weeks (60.1 ± 7.5% vs. 48.6 ± 6.1%, *P* < 0.05), and 12 weeks (63.0 ± 6.7% vs. 39.6 ± 9.8%, *P* < 0.01). The LVDs were significantly smaller in the hiPSC-CM patch group than in the sham group after 4 weeks (20.7 ± 2.6 mm vs. 28.3 ± 1.3 mm, *P* < 0.01), 8 weeks (21.0 ± 3.5 mm vs. 29.0 ± 5.8 mm, *P* < 0.05), and 12 weeks (21.6 ± 4.7 mm vs. 31.0 ± 3.1 mm, *P* < 0.05), whereas LVDd values did not significantly differ between the two groups. The echocardiography results showed improvement in LVEF and LVD, which indicated recovery of overall heart function.

To investigate regional changes in the heart function, a cardiac MRI was performed to compare the LV circumferential strain (CS) values at the preimplantation baseline and 12 weeks postimplantation (Fig. 3b, c). In the sham group, the CS levels of the LAD, left circumflex artery (LCx), and right coronary artery (RCA) regions did not significantly change after 12 weeks relative to those at baseline. In contrast, in the hiPSC-CM patch group, the CS levels in the LCx and RCA regions were significantly greater after 12 weeks compared to those at baseline (LCx: − 20.0 ± 7.3% vs. − 25.5 ± 7.3%, *P* < 0.05; RCA: − 18.4 ± 2.4% vs. − 20.8 ± 2.1%, *P* < 0.05), whereas CS levels in the LAD territory did not significantly change. The cardiac MRI results showed that cardiac function improvements occurred in the infarct-border zone rather than in the infarct zone.

Next, pathological interstitial fibrosis was assessed 12 weeks posttreatment using Masson's trichrome staining (Fig. 3d). The interstitial fibrosis area did not significantly differ between the hiPSC-CM patch and sham groups (*P* = 0.088).

**Fig. 4:**
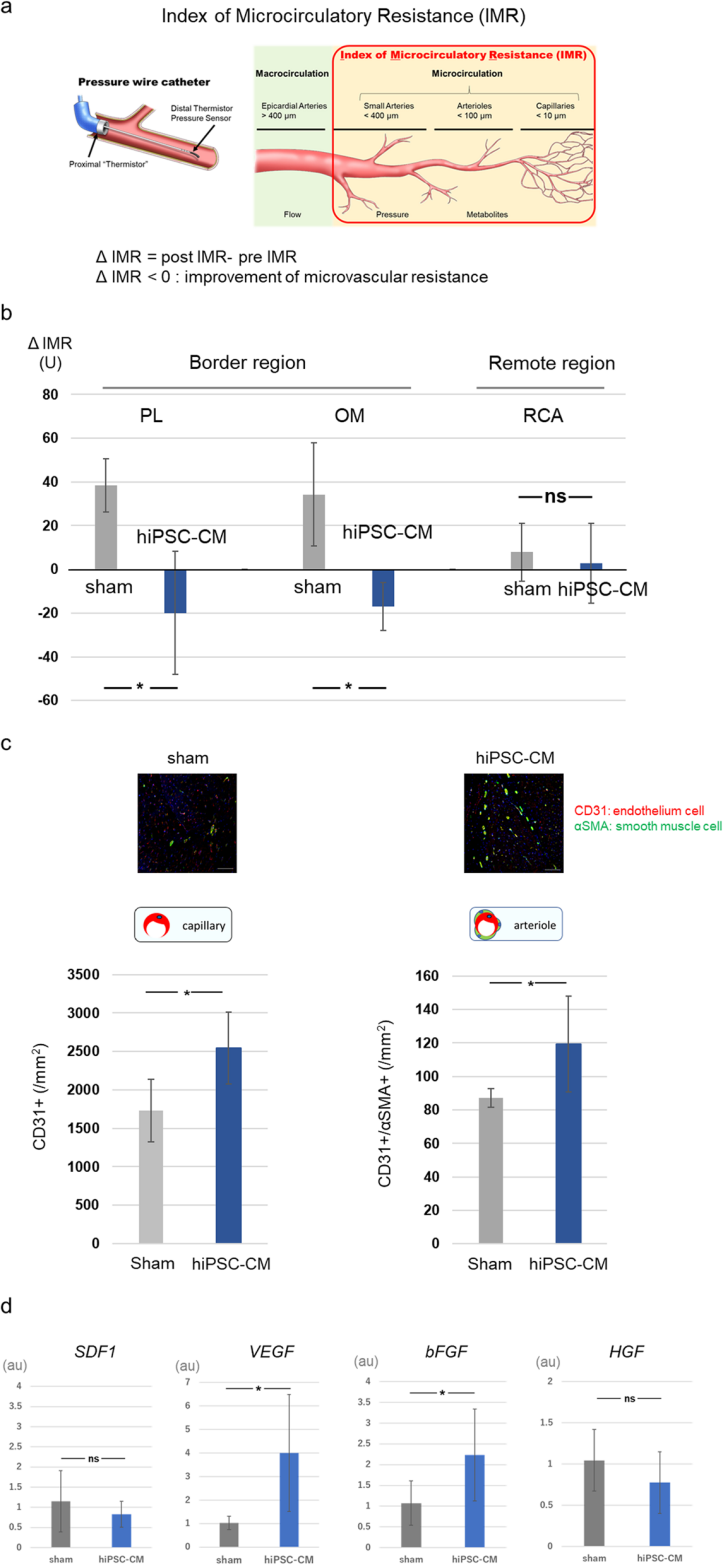
IMR and cytokine expression following transplantation of the hiPSC-CM patch in a porcine MI model. **a**, **b**: Schematic representation of IMR. ΔΙΜΡ was defined as IMR (postimplantation) − IMR (preimplantation). sham: n = 4, hiPSC-CM patch: n = 6 **c**: Upper panel, representative image of capillaries and arterioles immunostained with CD31 (red) and αSMA (green) at the infarct-border zone; lower panel, quantification of the number of CD31- and αSMA-positive cells. Each section was evaluated in 10 fields of view. Scale bar: 100 μm. sham: n = 4, hiPSC-CM patch: n = 6. **d**: Gene expression of proangiogenic factors in the infarct-border region 4 weeks following treatment. The myocardium around the infarct zone, which exhibited regional fibrosis, was excised and evaluated. Data are presented as the mean ± SD. Sham: n = 4, hiPSC-CM patch: n = 6, *P < 0.05

An angiogram and pressure wire study were also conducted to assess treatment-induced remodeling of the coronary artery branch network (Fig. 4a, b). The proximal LAD was completely occluded in all subjects. We defined the delta index of microvascular resistance (ΔIMR) as IMR (postimplantation)—IMR (preimplantation). ΔIMR in the LCx region (infarct-border zone), such as in the posterolateral branch (PL) (− 20.0 ± 28.2 vs. 38.4 ± 12.2, *P* < 0.05) and obtuse marginal branch (− 17.0 ± 11.1 vs. 34.3 ± 23.7, *P* < 0.05), was significantly lower in the hiPSC-CM patch group than in the sham group. The ΔIMR in the RCA region (infarct zone) was not significantly different between the two groups.

Vascular density of the infarct-border zone was assessed 12 weeks posttreatment using immunohistochemical analysis of CD31 and αSMA (Fig. 4c). The density of CD31-positive capillaries and CD31/αSMA double-positive arterioles was significantly greater in the hiPSC-CM patch group than in the sham group (2,542.2 ± 465.3/mm^2^ vs. 1,732.4 ± 405.2/mm^2^, *P* < 0.05; 119.4 ± 28.5/mm^2^ vs. 87.2 ± 5.5/mm^2^, *P* < 0.05, respectively; Fig. 4c). Moreover, transcript-level expression of proangiogenic cytokines in the infarct-border region was also assessed 4 weeks after treatment. VEGF and basic fibroblast growth factor (bFGF) expression levels were significantly higher in the infarct-border region of the hiPSC-CM patch group than in the infarct-border region of the sham group (VEGF: 4.00 ± 2.48 vs. 1.03 ± 0.28, *P* < 0.05; bFGF: 2.23 ± 1.10 vs. 1.07 ± 0.53). However, SDF-1 and HGF expression did not significantly differ between the two groups (Fig. 4d).

To assess the effects of the hiPSC-CM patch on the electrophysiology of the myocardium, the patch and sham groups were monitored via 24-h Holter electrocardiography 7 d prior to and 0, 1, 2, 3, 7, 14, 28, 42, 56, 70, and 84 d post-transplantation (Additional file 1: Table S7). No lethal arrhythmias, such as ventricular tachycardia and ventricular fibrillation, were observed during the study period. In addition, tumor formation was not detected during the study period (Additional file 1: Figure S8).

### Safety assessment for tumorigenicity and toxicity

To evaluate the safety of hiPSC-CM patches, general toxicity and tumorigenicity tests were performed. To assess general toxicity, a single hiPSC-CM patch was applied to the heart surface of male and female NOG mice and the mice were observed for 4 weeks. The general condition and body weight were recorded. Hematological and blood biochemistry tests and pathological assessments were also performed. No significant toxicity was observed following the application of the hiPSC-CM patch (Additional file 1: Table S8–S13).

**Fig. 5:**
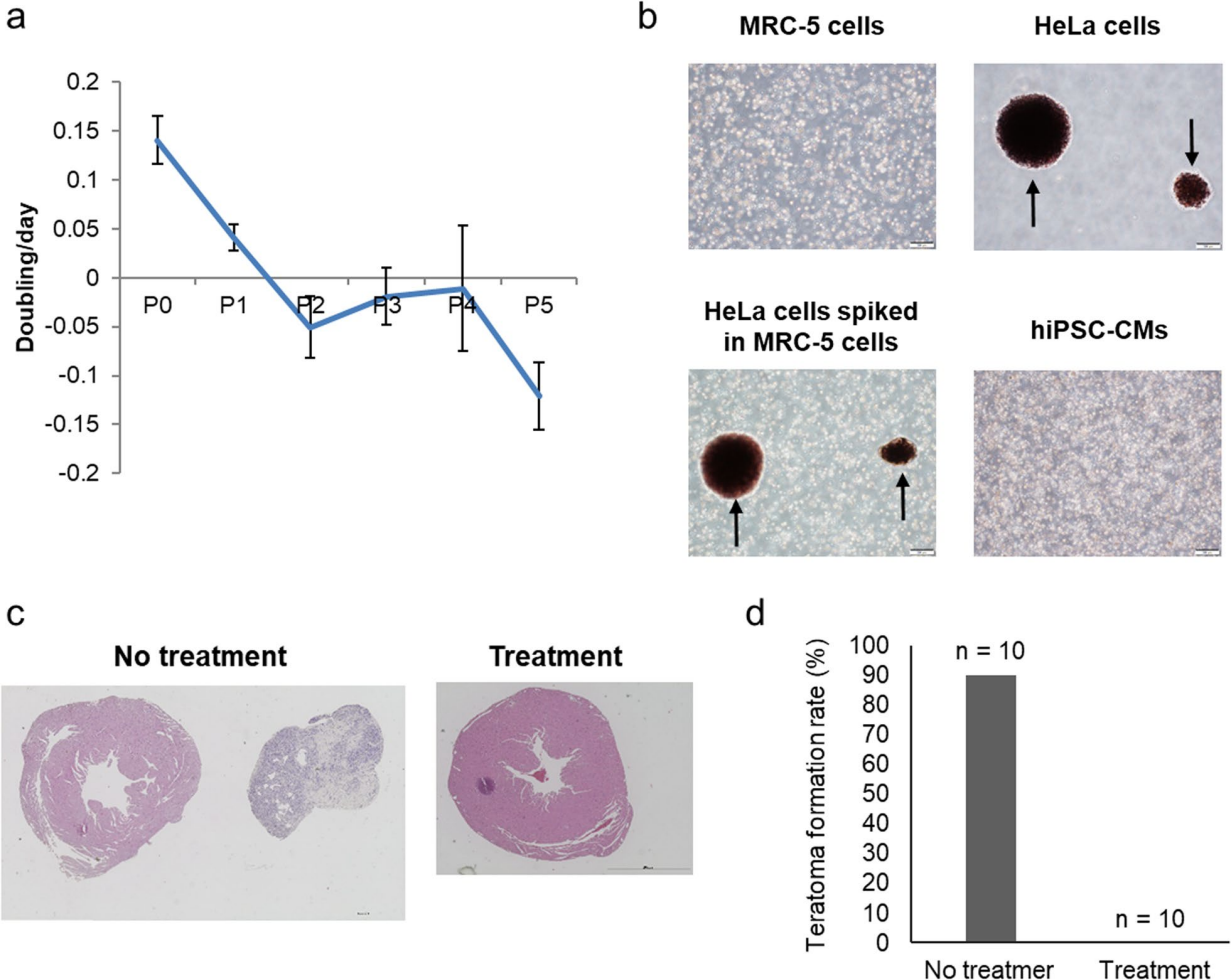
Detection of tumorigenic cells in vitro and in vivo. **a**: Cell growth assay of each passage. **b**: Soft agar colony formation assay. Phase-contrast micrographs of MRC-5 cells, HeLa cells, HeLa cells spiked into MRC-5 cells, and hiPSCs-CMs cultured on soft agar medium for 21 d. Arrows indicate colonies. Scale bar: 200 μm. **c**, **d**: Tumorigenicity was evaluated through transplantation of hiPSC-CM patches with or without purification of cardiomyocytes and elimination of residual undifferentiated hiPSCs into the left ventricular surface of immunodeficient NOG mice. **c** Representative hematoxylin and eosin staining of teratomas. Scale bar: 1000 µm (left panel) and 2000 µm (right panel). **d** Quantification of the rate of teratoma formation (no treatment group: hiPSC-CM patches without purification of cardiomyocytes and elimination of residual undifferentiated hiPSCs, n = 10; treatment group: hiPSC-CM patches with purification of cardiomyocytes and elimination of residual undifferentiated hiPSCs, n = 10)

To assess tumorigenicity, we monitored the animals for teratomas and malignant tumors caused by residual undifferentiated hiPSCs and malignantly transformed cells, respectively, using the in vitro and in vivo assays previously reported [14] to detect of potential tumorigenic cells in hiPSC-CMs. In vitro, we performed cell growth and soft agar colony formation assays, which are highly sensitive methods for detecting malignantly transformed cells, to identify cells that have undergone malignant transformation during hiPSC culture and cardiomyocyte differentiation. The growth rate of hiPSC-CMs during P5 was significantly lower than that during the initial passage (− 0.12 ± 0.04 vs. 0.14 ± 0.03 doubling/d, *P* < 0.01), suggesting that hiPSC-CMs contain no abnormal, excessively replicating cells (Fig. 5a). The soft agar colony formation assay showed no malignant growth of hiPSC-CMs (Fig. 5b). To detect teratoma-forming cells in the hiPSC-CM patch, the expression of Lin28A, a marker of undifferentiated hiPSCs, was examined in vitro (Additional file 1: Figure S2c). Lin28A expression was below the limit of detection in hiPSC-CMs 30 d after cardiac differentiation. Finally, we assessed tumorigenicity in vivo and found that transplantation of hiPSC-CM patches without purification of cardiomyocytes and elimination of residual undifferentiated hiPSCs induced tumor formation (nine out of ten mice) for 16 weeks following transplantation. In contrast, no teratomas or malignant tumors formed after transplantation of the hiPSC-CM patches with purification treatment (zero out of ten mice; Fig. 5c, d, Additional file 1: Table S8).

In addition to residual undifferentiated cells or malignantly transformed cells, critical genomic changes and the survival of foreign genes in hiPSCs may lead to tumor formation in hiPSC-CMs. Therefore, we performed whole-genome sequencing (WGS) and whole-exome sequencing (WES) of hiPSCs, hiPSC-CMs, and hiPSC-CM patches. No single-nucleotide variants (SNVs) or indels were found for the cancer-related genes listed in the Catalog of Somatic Mutations in Cancer (COSMIC) [15], the Cancer Gene Census (v79) [16], or Shibata’s list [17]. In addition, the identified mutations were not registered in the Human Gene Mutation Database Pro database (2016.4) [18]. We calculated variant allele frequencies (VAFs; Table 1) at SNV/indel positions found by Genomon [19] and Genomon2 [20] in the context of WGS and WES data. We also investigated copy number variations (CNVs) based on WGS and SNP array data. No CNVs were found in exons. The genome analyses are summarized in Table 2.

**Table 1:** Whole-genome sequencing results for hiPSCs (MCB), hiPSC-CMs, and hiPSC-CM patches

Sample	Number of SNVs/indels on CDS/splicing sites		Number of SNVs/indels on CDS/splicing sites (Census/Shibata’s list)	Number of CNVs on exons	Number of CNVs on exons (Census/Shibata’s list)
	WGS	WES	WGS/WES	WGS/SNP array	WGS/SNP array
MCB	15	11	0	0	0
MCB (expansion culture)	14	11	0	0	0
hiPSC-CMs	14	12	0	0	0
hiPSC-CM patch	14	11	0	0	0

CNV, copy number variation; hiPSC-CM, human induced pluripotent stem cell-derived cardiomyocyte; MCB, master cell bank; SNP, single-nucleotide polymorphism; SNV, single-nucleotide variant; Indels, insertions/deletions; WGS, whole-genome sequencing; WES, whole-exome sequencing

**Table 2:** Variant allele frequencies as per the called SNVs/indels

Chr	Start	End	Ref	Alt	Func refGene	Gene refGene	MCB		MCB (expansion culture)		hiPSC-CM		hiPSC-CM patch	
							cov	alt_ ratio	cov	alt_ ratio	cov	alt_ratio	cov	alt_ ratio
chr3	78,708,860	78,708,860	T	A	exonic	*ROBO1*	59	64.4%	61	54.1%	39	56.4%	60	56.7%
chr5	83,356,228	83,356,228	G	A	exonic	*EDIL3*	96	45.8%	98	37.8%	58	55.2%	82	46.3%
chr5	175,837,271	175,837,271	C	G	exonic	*CLTB*	81	46.9%	77	46.8%	58	39.7%	68	41.2%
chr7	100,175,865	100,175,865	A	G	exonic	*LRCH4*	65	46.2%	97	45.4%	63	58.7%	87	39.1%
chr8	4,851,937	4,851,937	A	T	exonic	*CSMD1*	52	53.8%	34	41.2%	59	47.5%	50	50.0%
chr9	103,340,555	103,340,555	G	A	exonic	*MURC*	66	54.5%	93	48.4%	75	50.7%	78	50.0%
chr11	48,286,119	48,286,119	C	T	exonic	*OR4X1*	82	50.0%	93	54.8%	68	55.9%	76	57.9%
chr20	1,300,304	1,300,304	C	T	splicing	*SDCBP2*	66	43.9%	63	46.0%	49	46.9%	48	43.8%
chr12	6,675,434	6,675,434	C	T	exonic	*NOP2*	83	21.7%	77	53.2%	53	56.6%	65	56.9%
chr12	50,746,672	50,746,672	A	G	exonic	*FAM186A*	98	30.6%	83	21.7%	58	17.2%	91	29.7%
chr15	42,511,798	42,511,798	T	A	exonic	*TMEM87A*	146	24.0%	145	1.4%	67	0.0%	110	0.9%
chr18	14,852,387	14,852,387	G	T	exonic	*ANKRD30B*	87	25.3%	100	2.0%	68	1.5%	116	2.6%
chr1	201,180,485	201,180,485	A	G	exonic	*IGFN1*	119	33.6%	139	26.6%	96	30.2%	117	40.2%
chr11	65,480,401	65,480,401	C	T	exonic	*KAT5*	61	24.6%	62	0.0%	59	1.7%	49	0.0%
chr8	23,115,566	23,115,566	G	A	exonic	*CHMP7*	87	12.6%	80	3.8%	63	1.6%	63	0.0%
chr10	114,182,146	114,182,146	G	T	exonic	*ACSL5*	93	1.1%	84	51.2%	53	47.2%	71	47.9%
chr17	33,749,201	33,749,203	TCT	-	exonic	*SLFN12*	105	0.0%	100	44.0%	70	37.1%	67	44.8%
chr1	152,883,009	152,883,009	T	C	exonic	*IVL*	49	12.2%	53	15.1%	72	11.1%	55	9.1%
chr16	15,711,240	15,711,240	G	A	exonic	*KIAA0430*	85	1.2%	102	12.7%	64	10.9%	68	7.4%
chr12	50,746,414	50,746,414	T	G	exonic	*FAM186A*	97	28.9%	82	29.3%	58	25.9%	82	36.6%
chr1	186,276,394	186,276,394	T	G	exonic	*PRG4*	46	17.4%	34	11.8%	52	28.8%	55	23.6%
chr3	195,512,734	195,512,734	T	G	exonic	*MUC4*	188	13.8%	175	13.1%	106	22.6%	151	15.9%
chr1	238,048,745	238,048,745	G	T	exonic	*ZP4*	92	0.0%	68	4.4%	51	5.9%	77	3.9%

alt_ratio, alternative ratio; Chr, chromosome; cov, depth of coverage; hiPSC-CM, human induced pluripotent stem cell-derived cardiomyocyte; Indels, insertions/deletions; MCB, master cell bank; SNV, single-nucleotide variant

## Discussion

In this study, we reported the biological characterization, efficacy, and safety of clinical-grade hiPSC-CM patches as part of a preclinical study. cTNT was present in 60–80% of the hiPSC-CMs, whereas the level of Lin28A, which is a marker of undifferentiated hiPSCs, was below the limit of quantitation. The safety of hiPSC-CM patches for clinical applications was confirmed through genomic analysis, in vitro cell growth assays, soft agar colony formation assays, and undifferentiated cell assays. Moreover, in vivo tumorigenicity and general toxicity tests were performed in immunodeficient mice, and an arrhythmia test was performed in a porcine MI model. Finally, an efficacy study using a porcine MI model demonstrated that hiPSC-CM patches ameliorated the distressed myocardium in terms of improved cardiac function and angiogenesis.

We established a clonal MCB of clinical grade hiPSCs and differentiated this MCB into cardiomyocytes. Since MCBs and hiPSC-CMs that had been evaluated for safety and quality in advance were stored, we could provide them promptly as needed, saving time and cost compared to those for autologous transplantation.

Although the tumorigenicity of undifferentiated hiPSCs, which is one of the safety evaluations, can be adequately verified through Lin28A expression and controlled by adminstration of brentuximab vedotin, which induces apoptosis of CD30-positive undifferentiated hiPSCs, malignantly transformed cells in hiPSC-CMs remain a major obstacle in clinical translation [14, 21]. Thus, we performed a genomic analysis to confirm the tumorigenicity of hiPSC-CM patches. Although no abnormal mutations were found, it remains undetermined as to which genomic mutations should be assessed to ensure safety other than those reported to be tumorigenic in oncogenomic databases such as COSMIC [15, 16] and Shibata’s list [17]. Numerous genetic mutations have been identified in living cells. Therefore, it is difficult to determine whether all mutations of potentially tumorigenic genes result in oncogenesis or whether some well-known tumorigenicity-associated genes, such as c-myc, can be ignored. Consequently, the relationship between genomic abnormalities and tumorigenicity in cell therapy has not been fully elucidated, and further studies are warranted to confirm the safety of these cells in terms of tumorigenicity. Nevertheless, in immunodeficient NOG mice, no malignantly transformed cells or tumorigenicity was observed.

Revealing the tumorigenicity of residual undifferentiated hiPSCs and verifying the presence of tumorigenic cells generated by the transformation of hiPSC-CM constructs are necessary. Lin28A expression levels correlate with the frequency of tumor formation in NOG mice [14]. Quantifying Lin28A levels may thus be an alternative to performing tumorigenicity tests of NOG mice when validating the safety of hiPSC-CMs in clinical applications.

In this preclinical study, no lethal arrhythmias were observed when hiPSC-CM patches were transplanted into the epicardium of the porcine heart. Patilla et al. [22] transplanted myoblasts into a rat heart failure model via intramyocardial injection or cell sheet transplantation and reported significant improvement in cardiac function in both groups. Arrhythmia was not observed after cell sheet transplantation, however, it was observed within two week of intramyocardial injection. Our results also showed no arrhythmia in the early phase or 12 weeks posttransplant. These results suggest that the main cause of arrhythmia may be the cell transplantation method and not the cell source. Thus, the hiPSC-CM patch could prevent tumorigenicity and arrhythmogenicity, suggesting that it is a clinically safe method for cell delivery. A clinical study is warranted to verify the safety of this treatment.

We have previously reported that compared with mesenchymal stem cell or skeletal myoblast transplantation, hiPSC-CM patch transplantation improved the recovery of cardiac function [23]. An important property of the hiPSC-CM patches is their electrical integration into a recipient heart with a low number of cardiomyocytes. Previous studies have shown that transplanted cardiomyocyte patches contract and relax synchronously with the recipient’s heart [4, 24]. We previously measured the contraction of the myocardium with high-intensity synchrotron radiation 2 weeks after iPSC-CM patch transplantation into rats and reported that the contraction cycle was synchronized with the host heart [4]. Additionally, we used fluorescent probe analyses to verify that the iPSC-CM patch was electrically synchronized with the host myocardium 5 d following transplantation into MI model rats [24]. Previous studies have also demonstrated that cardiomyocyte patches exhibit excellent cardiogenic properties, cardiomyogenesis potential, and angiogenic ability [25–27]. In this study, the patch was not discernable when the animal was sacrificed 12 weeks post-hiPSC-CM patch transplantation. Our previous report confirmed that hiPSC-CM patch transplanted in a porcine MI model survived until 8 weeks [25]. Therefore, it is considered that the transplanted cells remained until at least 8 weeks after transplantation. We previously investigated cell survival following allogenic transplantation of an iPSC-CM patch into cynomolgus monkeys [28]. When the major histocompatibility complex (MHC)-mismatched cynomolgus macaque iPSC-CM patch was transplanted into the heart in a cynomolgus macaque model, the graft survived for up to 3 months under immunosuppressive drug administration. During the time the graft was observed, cardiac function improved and was maintained, possibly owing to the induction of angiogenesis through paracrine signaling.

Angiogenesis may have a positive impact on hibernating myocardium, initiating the functional recovery of the heart [25–27]. MI in the LAD region led to cardiac function deterioration. Additionally, the infarct-border zone also underwent remodeling, resulting in cardiomyocyte hypertrophy and interstitial fibrosis, causing cardiac function deterioration. Cardiac function was considered unlikely to improve in the LAD region owing to fibrosis; however, myocardial viability improved in the infarct-border zone. Histological analysis suggested that the LAD region is characterized by the recognition of functional blood vessels with smooth muscle cells lining vascular endothelial cells [25–27]. In particular, cytokines of the angiopoietin family may be enriched in vitro. Angiopoietins greatly contribute to the maturation of blood vessels. In general, ischemic cardiomyopathy is characterized by myocardial ischemia arising from the disruption and stenosis of the vasculature network in coronary arteries [25–27]. Here, the heart failure model animals transplanted with hiPSC-CM patches demonstrated low peripheral coronary vascular resistance, which was likely due to the maturation of blood vessels and opening of the occluded peripheral vascular network. In addition, we could only conduct experiments on female pigs. Therefore, the effects of estrogen and other hormones on cardioprotection must be taken into consideration. However, a consistent degree of cardiac dysfunction was observed in the MI model, suggesting that estrogen may have little effect on cardiac function among females.

Further studies are required to assess the effects of cell-based regenerative myocardial tissue on cardiac function. We have previously reported that poly (d,l-lactic-co-glycolic acid) (PLGA) nanofibers can be used to create an aligned tissue of myocardial cells [29]. However, a safety test of PLGA nanofibers is lacking, and hence, no approval for clinical use has been obtained. Demonstrating the safety of PLGA nanofibers is necessary for the use of this material in the treatment of cardiac dysfunction in the future.

## Conclusions

In this study, we verified that a clinical-grade hiPSC-CM patch could function as a feasible, safe, and effective myocardial tissue in a preclinical study. Further translational research via a clinical trial on allogenic hiPSC-CM patches for patients with ischemic heart failure is warranted.

### Supplementary Information

**Additional file 1**. Supplemental methods. Supplemental tables (Tables S1–S13). Supplemental figures (Figure S1–S8). Supplemental references.

## Data Availability

Most data generated or analyzed during this study are included in this manuscript and its supplementary information files or are available from the corresponding author on a reasonable request. The single-cell RNA-seq datasets generated and analyzed during the current study are available in the Japanese Genotype-phenotype Archive repository, Study number: JGAS000665. The whole-genome sequencing datasets generated and analyzed during the current study are available in the European Genome-phenome Archive (EGA), EGA Dataset Accession ID: EGAD50000000274.

## Abbreviations

ΔIMR: Delta index of microvascular resistance
bFGF: Basic fibroblast growth factor
α-MHC: Alpha cardiac myosin heavy chain
β-MHC: Beta cardiac myosin heavy chain
CNVs: Copy number variations
COSMIC: Catalogue of somatic mutations in cancer
CS: Circumferential strain
cTNT: Cardiac troponin T
DMEM: Dulbecco’s modified eagle medium
ECM: Extracellular matrix proteins
FBS: Fetal bovine serum
GMP: Good manufacturing practices
HGF: Hepatocyte growth factor
hiPSCs: Human induced pluripotent stem cells
hiPSC-CM: Human induced pluripotent stem cell-derived cardiomyocyte
LAD: Left anterior descending artery
LCx: Left circumflex artery
LV: Left ventricle
LVDd: LV end-diastolic diameter
LVDs: LV end-systolic diameter
LVEF: LV ejection fraction
MCB: Master cell bank
MLC2a: Atrial isoform of the myosin light chain 2
MLC2v: Ventricular isoform of myosin light chain
NOG: NOD/Shi-scid, IL-2R γ^null^
P: Passage
PL: Posterolateral branch
RCA: Right coronary artery
SNV: Single-nucleotide variants
STR: Short tandem repeat
VAFs: Variant allele frequencies
VEGF: Vascular endothelial growth factor
WES: Whole-exome sequencing
WGS: Whole-genome sequencing
