# Supplementary material for: Pre-clinical evaluation of the efficacy and safety of human induced pluripotent stem cell-derived cardiomyocyte patch
Source: Stem Cell Res Ther. 2024 Mar 13;15:73. doi: 10.1186/s13287-024-03690-8 (PMC10935836; doi:10.1186/s13287-024-03690-8)
Supplement: Supplementary file 1 — Additional file 1. Supplemental methods. Supplemental tables (Tables S1–S13). Supplemental figures (Figure S1–S8). Supplemental references. [file 13287_2024_3690_MOESM1_ESM.docx]

Additional files

**Pre-clinical evaluation of the efficacy and safety of human induced pluripotent stem cell-derived cardiomyocyte patch**

Page S2 – Supplemental methods

Page S24 – Supplemental tables (Tables S1–S13)

Page S46 – Supplemental figures (Figure S1–S8)

Page S61 – Supplemental references

**Supplemental methods**

**Cardiomyogenic differentiation, purification, and elimination of residual undifferentiated human induced pluripotent stem cells (hiPSCs)**

The hiPSC line QHJI14s04 was cultured on iMatrix511 (Nippi, Tokyo, Japan)-coated dishes in Stem Fit Ak03N (Ajinomoto, Tokyo, Japan), followed by cardiac differentiation, purification, and elimination of residual undifferentiated hiPSCs. An aliquot of the cells was also frozen. Cardiomyogenic differentiation of QHJI14s04 cells was induced as previously described [1]. In brief, for cardiomyogenic differentiation, we generated embryoid bodies (EBs) from hiPSCs and cultured them in bioreactors (capacity, 100 mL; Able Corp., Tokyo, Japan) with various recombinant proteins and chemicals for 16 d. After differentiation, cardiomyocyte aggregates were cultured in glucose-free Dulbecco’s Modified Eagle Medium (DMEM) (Thermo Fisher Scientific, Waltham, MA, USA) for 7 d for purification, and cardiomyocytes were dissociated as previously described [2]. The dissociated cells were cultured at a density of 1.7 × 10^6^ cells/cm^2^ in DMEM (Nacalai Tesque, Kyoto, Japan) supplemented with 10% fetal bovine serum (FBS) (Sigma‒Aldrich, St. Louis, MO, USA) and 10 μg/mL brentuximab vedotin (ADCETRIS™, Takeda, Osaka, Japan) at 37 ℃ under 5% CO_2_ for 5 d to eliminate residual undifferentiated cells [3]. The cells were suspended in a cell banker (Nippon Genetics, Tokyo, Japan) and stored at −80 ℃ using a programmed freezer (FZ2000; STREX Inc. Osaka, Japan).

**Immunofluorescence**

The cardiomyocyte aggregates, hiPSC-cardiomyocyte (hiPSC-CM) patches, and excised heart samples were fixed in 4% paraformaldehyde, frozen in liquid nitrogen, and cryosectioned. Immunofluorescence was performed using the primary and secondary antibodies listed in Table S1. Cell nuclei were counterstained with Hoechst 33342 (1:100; Dojindo, Kumamoto, Japan) and imaged using a confocal laser scanning microscope (FV10i; Olympus, Tokyo, Japan). The system was controlled using FV10-ASW 3.1 software (Olympus).

**RNA isolation and quantitative polymerase chain reaction (qPCR)**

Total RNA was extracted from the cells and heart tissues using the RNeasy Mini Kit (Qiagen, Hilden, Germany) and reverse-transcribed into cDNA using the SuperScript VILO cDNA Synthesis kit (Thermo Fisher Scientific). qPCR was performed with the ViiA 7™ Real-Time PCR or ABI PRISM 7700 systems (Applied Biosystems, Foster City, CA, USA) using either SYBR Green (Applied Biosystems) or TaqMan™ probes (Applied Biosystems). The primer sequences used in this study are listed in Table S2. Each sample was analyzed in triplicate. The expression of target genes was normalized to that of glyceraldehyde-3-phosphate dehydrogenase (*GAPDH*) as the control. Relative gene expression was determined using the 2^−ΔΔCt^ method. The data were analyzed using the software provided along with the ViiA 7™ Real-Time PCR or ABI PRISM 7700 systems.

**PCR arrays**

Total RNA was extracted from the hiPSCs and hiPSC-CMs using the RNeasy Mini Kit. Total RNA from human adult and fetal heart samples were purchased from Takara Bio (Kusatsu, Japan). To analyze human stem cell-associated genes, cDNA was synthesized using the RT^2^ First Strand Kit (Qiagen). The ViiA 7™ Real-Time PCR system was used to run the RT² Profiler™ PCR Array Human Stem Cell array (Qiagen). Cluster analysis was performed using the online RT^2^ Profiler™ PCR Array software provided by SABiosciences (freely available from https://dataanalysis.qiagen.com/pcr/arrayanalysis.php?target=plothome). To analyze cardiac differentiation-associated genes, cDNA was synthesized using the SuperScript VILO cDNA Synthesis Kit (Thermo Fisher Scientific), and gene expression was analyzed using the TaqMan™ Array Human Cardiomyocyte Differentiation by BMP Receptors (Thermo Fisher Scientific).

**Flow cytometry**

After fixation in Fixation/Permeabilization solution (BD Biosciences, Franklin Lakes, NJ, USA), cells were probed with the following antibodies: anti-cTNT (1:300; sc-20025; Santa Cruz Biotechnology, Dallas, TX, USA), anti-αSMA (1:100; ab32575; Abcam, Cambridge, UK), anti-vimentin (1:100; ab92547; Abcam), and anti-CD31 (1:10; 561654; BD Biosciences), followed by incubation with fluorescently conjugated secondary antibodies. Cell populations were resolved using the FACSCanto II system (BD Biosciences). Data were analyzed using Diva (BD Biosciences) and FlowJo (TreeStar Inc., Ashland, OR, USA) software.

**Single-cell preparation and single-cell RNA-seq**

The single-cell sequencing library was generated using the ICELL8 cx platform (Takara Bio). In brief, isolated cells were stained with a mixture of Hoechst 33342 and propidium iodide (R37610; Thermo Fischer Scientific) following the manufacturer’s instructions. After staining, the cells were washed with phosphate-buffered saline (PBS) and counted using a hemocytometer. The cell suspension was then pipetted into a 384-well plate, and then the solution was dispensed onto 250 nL ICELL8 cx system chips (640199; Takara Bio). Imaging and analysis of the nanowells were conducted using CellSelect Software, and individual live cells, defined by Hoechst-positive and propidium iodide-negative staining, were selected. After dispensing the MasterMix (640167; Takara Bio) into the selected nanowells, the chip was sealed, centrifuged, and placed into a Chip Cycler (Bio-Rad Laboratories, Hercules, CA, USA) for reverse transcription and synthesis of full-length cDNA, as per the manufacturer’s protocols. The resulting cDNAs were purified, and fragments up to 300–350 bp in size were removed using 0.6× Agencourt AMPure XP beads (A63880; Beckman Coulter, Brea, CA, USA). One nanogram of the pooled cDNA was used as an input to generate a sequencing library using the Nextera XT DNA sample preparation kit (FC-131-1024; Illumina, San Diego, CA, USA), as per the manufacturer’s protocols. Libraries were sequenced on a HiSeq 3000 sequencer (Illumina) using the 100 bp paired-end sequencing protocol.

For single-cell RNA-seq analysis, raw reads were processed using the mappa/hanta pipeline (Takara Bio), and the Seurat R package (v3.1.5) [4] was used to perform further feature selection and clustering. Single cells with over 200 expressed genes were selected. We clustered the single cells by identifying the top 2,000 highly variable genes and performed a principal component analysis based on the scaled expression values by total expression, followed by graph-based cluster detection using the top 10 principal components. Single cells were represented in a two-dimensional uniform manifold approximation and projection plane, whereas clusters were annotated according to the marker gene composition.

**Intracellular calcium imaging of hiPSC-CMs**

The hiPSC-CMs were seeded onto 96-well plates at a density of 1–2 × 10^5^ cells/well. Intracellular calcium imaging analysis was performed as previously described [5]. Cells were loaded with 2 µM Cal-520™ (AAT Bioquest, Sunnyvale, CA, USA) in PBS for 2 h at 37 °C. Next, the loading buffer was replaced with the culture medium. The basal activity was recorded using an FDSS/µCELL (Hamamatsu Photonics, Shizuoka, Japan). Subsequently, various concentrations of isoproterenol (0.1, 1, 10, 100, and 1,000 nM; Merck) or E-4031 (Merck) were added to the culture medium, and cells were monitored for 30 min after the addition of each drug. Parameters such as the peak rate, peak-to-peak time, upstroke slope, downstroke slope, and 90% peak-width duration (PWD90) were calculated from the ratio of fluorescence intensity before and after the treatment to characterize the intracellular calcium levels. Data are expressed as the mean ± SD.

**Contraction properties of hiPSC-CMs**

hiPSC-CMs were seeded into 96-well plates at a density of 1–2 × 10^5^ cells/well. Cell motion analysis was performed as previously described [5]. The motion was recorded using the following parameters: frame rate, 150 fps; depth, 8 bits; resolution, 1,024 × 1,024 pixels with a Cell Motion Imaging System (SI8000; Sony Biotechnology, Tokyo, Japan). Subsequently, various concentrations of isoproterenol (0.1, 1, 10, 100, and 1,000 nM; Merck) or E-4031 (Merck) were added to the culture medium, and the cells were monitored for 30 min after the addition of each drug. Data on arrhythmia-like abnormal contractions after the addition of E-4031 were excluded from the analysis, as they could not be compared with data on contractions at regular intervals. The relative change in each parameter, such as beating rate, peak interval, contraction/relaxation velocity, and contraction relaxation duration (CRD) after drug administration, was calculated using the predrug treatment samples as the control group. Data are expressed as the mean ± SD.

**Electron microscopy**

Cardiac tissues were prefixed with Karnovsky fixative (2.5% glutaraldehyde and 2% paraformaldehyde in 0.1 M cacodylate buffer [pH 7.4]) for 2 h at 4 °C and postfixed with 2% osmium tetroxide (Nisshin EM, Tokyo, Japan) for 2 h at 4 °C. The samples were then immersed in 0.5% uranyl acetate (Fujifilm Wako Pure Chemical Corporation, Osaka, Japan) for 3 h at room temperature, dehydrated in an ethanol gradient (50, 70, 80, 90, 95, and 100%; Muto Pure Chemicals, Tokyo, Japan) and propylene oxide (Sigma‒Aldrich), and embedded in epoxy resin. Semithin sections (0.5 μm) were stained with 0.1% toluidine blue (Merck, Darmstadt, Germany) and examined under a light microscope. Ultrathin sections were prepared using an EM UC7 ultramicrotome (Leica Microsystems, Wetzlar, Germany). These sections were counterstained with uranyl acetate and lead citrate before examination using an H-7500 electron microscope (Hitachi High-Technologies, Tokyo, Japan) at 80 kV.

**Measurement of cytokine levels**

The supernatants of cell patches were collected after culturing under normoxic or hypoxic (5% O_2_) conditions and analyzed using a fluorescence-dyed microsphere-based immune assay (Bio-Rad Laboratories) according to the manufacturer’s instructions. The concentrations of cytokines, such as angiogenin, angiopoietin-1, angiopoietin-2, HGF, and VEGF, were measured and analyzed using the Bioplex suspension array system (Bio-Rad Laboratories).

**Electrophysiological properties of the hiPSC-CM patches**

The hiPSC-CM patches seeded on a 96-well plate were transferred onto an MED probe (Alpha MED Scientific, Osaka, Japan) and incubated for 3–5 days until they were attached to the probe. The spontaneous beating of hiPSC-CM patches was confirmed under a microscope and measured. Extracellular field potentials were monitored using a multielectrode array system (MED64; Alpha MED Scientific), recorded for 10 min, and analyzed using Mobius software (Alpha MED Scientific).

**Mechanical properties of the hiPSC-CM patches**

Mechanical properties were assessed using the MicroTester G2 (CellScale, Waterloo, ON, Canada). The hiPSC-CM patches were transferred into a heated bath containing culture medium at 37 °C. The two ends of each hiPSC-CM patch were hooked to a fixed wire and a force-sensing probe. The force generated by the patch was calculated based on the displacement of the probe. The probe was moved downward to stretch the patch to different extents until the tissue broke. The force data were recorded at a sample rate of 5 Hz.

**Generation of the porcine chronic myocardial infarction model and hiPSC-CM patch transplantation**

The experimental protocol for investigating the efficacy of the hiPSC-CM patch using a porcine myocardial infarction (MI) model is shown in Figure S7a. The sample size was determined from a previous experiment conducted by our group [7]. A chronic MI model was generated by placing an ameroid constrictor (COR-4.0-SS; Research Instruments SW, Escondido, CA, USA) around the proximal left anterior descending coronary artery (LAD) and ligation of the distal LAD after the second diagonal branch [6] in healthy 7–8-month-old mature female Clawn minipigs (Kagoshima Miniature Swine Research Center, Kagoshima, Japan) weighing 20–25 kg (Figure S7b). Four of the 18 minipigs died within four weeks after LAD ligation. Four weeks after the procedure, we selected the minipigs with successful chronic heart failure modelling (left ventricular ejection fraction [LVEF] <50%) using cardiac echocardiography and cardiac MRI (Figure S7c). The minipigs were randomly divided into two groups using a computer-based random order generator: one group comprised those undergoing hiPSC-CM patch transplantation (hiPSC-CM patch group; n = 7) and the other group comprised those undergoing sham operation (sham group; n = 4). During the procedure, three of the sham group minipigs died before transplantation due to anesthesia, and we decided to perform the sham group with four animals. Animals in the sham group were subjected to the same surgical procedure, except for the cell patch placement. In the hiPSC-CM patch group, two patches containing 1 × 10^8^ cells were transplanted onto the infarcted myocardium to cover the infarct and surrounding border areas.

The transplanted cell patches were attached to the surface of the heart, fixed with 6-0 prolene sutures, and sprayed with fibrin glue (Beriplast P; CSL Behring, King of Prussia, PA, USA) to prevent movement (Figure S7d, e). The hiPSC-CM patches were transplanted under general anesthesia; tacrolimus was also administered (5 mg) orally. As transplanted cells were derived from human tissue, all animals received immunosuppressive drugs, such as tacrolimus (0.75 mg/kg; Astellas Pharma Inc., Tokyo, Japan), methylprednisolone (20 mg; Takeda Pharmaceutical Co. Ltd, Osaka, Japan), and mycophenolate mofetil (500 mg; Teva Czech Industries sro., Opava, Czechia) [7], which were administered daily starting 5 d before transplantation until euthanasia. The hiPSC-CM patches were transplanted via median sternotomy under general anesthesia by inhalation of 2% isoflurane (Fujifilm) and continuous injection of 6 mg/kg/h propofol (Diprivan; AstraZeneca, Osaka, Japan).

After surgery, the minipigs were allowed to recover in individual temperature-controlled cages (20–22 °C). Later, they were humanely euthanized via the intravenous administration of potassium chloride (1–2 mmol/kg) under deep anesthesia with inhalation of 5% isoflurane for analysis according to the Osaka University Regulations on Animal Experiments. The hearts were immediately harvested.

**Cardiac echocardiography**

Transthoracic echocardiography was performed under general anesthesia using a 5.0 MHz transducer (Aplio Artida; Toshiba Medical Systems, Tokyo, Japan). The left ventricular end-diastolic (LVEDV) and end-systolic (LVESV) volumes were calculated using the Teichholz formula [8]. The LVEF was calculated as follows: LVEF (%) = 100 × (LVEDV − LVESV)/(LVEDV).

**Cardiac catheter**

Fluoroscopy-guided selective coronary angiography was performed by injecting iopamidol through a catheter (Vista Brite Tip; Cordis, Miami Lakes, FL, USA) inserted from the right femoral artery in the supine position under general anesthesia. A fluoroscopy-guided pressure wire (Radi Medical Systems, Uppsala, Sweden) was inserted to assess the myocardial microvascular resistance in the left circumflex coronary artery (LCx) (posterolateral and obtuse marginal branches) and right coronary artery (RCA) territories, as described by Fearon *et al.* [9]. The coronary pressure wire was calibrated outside the body, equalized to the pressure reading from the guide catheter with the pressure sensor positioned at the ostium of the guide catheter, and then advanced to the distal two-thirds of the LCx and RCA. The index of microvascular resistance (IMR) was determined as follows: 3 mL of room temperature saline was injected into the cardiac catheter three times at rest, and the resting transit times were recorded and averaged. Maximal hyperemia was then achieved using continuous intravenous adenosine at 180 mg/kg/min via a venous catheter. The maximal hyperemic transit time was measured three times and averaged. The mean aortic and distal coronary pressures were recorded during peak hyperemia. An IMR ≤ 25 was considered normal [10].

**Cardiac MRI**

Cardiac MRI was performed under general anesthesia using a 1.5-T MR scanner (SIGNA EXCITE XI TwinSpeed; GE Medical Systems, Milwaukee, WI, USA) immediately before and 12 weeks after transplantation. The images were analyzed using 2D CPA MR (Tom-Tec Imaging Systems, Unterschleissheim, Germany), a commercial feature-tracking vector-based analysis tool based on a hierarchical algorithm that has been previously validated in clinical studies [11]. For each of the three short-axis plane cine images, the left ventricle endocardial border at the end-diastolic frame was manually drawn on a single frame by an expert reader. The software then automatically propagated the contour and followed its features throughout the cardiac cycle to draw the circumferential strain of the 17 segments, as per the American Heart Association model. The 17 segments were compiled into three territories according to coronary artery domination: LAD (territories 1, 2, 7, 8, 13, and 14), LCx (territories 5, 6, 11, 12, and 16), and RCA (territories 3, 4, 9, 10, and 15).

**Telemetered Holter electrocardiography**

To assess safety with respect to arrhythmias, we recorded 24-hour electrocardiograms using a Holter recorder (PhysioTel Digital, DSI, St. Paul, MN, USA) 7 d before and on days 0, 3, 7, 14, 28, 42, 56, 70, and 84 after implantation. The electrodes were placed in the chest and connected to the Holter recorder. The radiofrequency signals were monitored and saved on a personal computer near the cages using a PhysioTel Digital System (DSI). Data were analyzed using HEM data analysis software (Notocord Systems SAS, Croissy-sur-Seine, France).

**Histological analysis**

All autopsy tissue specimens of murine and porcine hearts transplanted with hiPSC-CM patches were fixed in 10% buffered formalin (Fujifilm) and embedded in paraffin using a Microm STP 120 Spin Tissue Processor (STP120-3; Thermo Fisher Scientific). Serial paraffin-embedded sections were sectioned at a thickness of 0.5 μm using a Microm HM 430 system (MIC 990010; Thermo Fisher Scientific), deparaffinized in xylene (Fujifilm), dehydrated in a graded series of ethanol (Fujifilm), and stained with hematoxylin and eosin (H&E; Muto Pure Chemicals). The sections were then imaged using a light microscope (DM4000B; Leica). To analyze fibrosis, paraffin-embedded sections were stained with Masson’s trichrome and imaged under a microscope. The percentage of the fibrotic area in the entire tissue was measured using MetaMorph software for Windows (Universal Imaging Corporation, Downingtown, PA, USA). Immunostaining was performed using an anti-CD31 antibody (1:50; Abcam), mouse anti-SMA antibody (1:50; Dako, Glostrup, Denmark), anti-Ki-67 antibody (1:100; Dako), and anti-lamin antibody (1:250; Abcam) (Table S1). Briefly, deparaffinized, dehydrated tissue sections were processed for antigen retrieval by autoclaving in 0.01 M citrate buffer (Dako). The sections were immersed in methanol (Fujifilm) containing 3% hydrogen peroxide (Fujifilm) and incubated overnight at 4 °C with the indicated primary antibodies. Subsequently, the sections were incubated with a biotinylated anti-mouse IgG antibody (K0675; Dako) and further incubated with peroxidase-conjugated streptavidin (Dako). Thereafter, the sections were visualized using DAB solution (Fujifilm) and imaged under a light microscope (Leica). Vascular density was measured at the infarct border zone in 10 randomly selected fields using a Biorevo BZ-9000 fluorescence microscope (Keyence; Osaka, Japan).

**Fluorescence *in situ* hybridization**

One week after transplantation, hiPSC-CM patches were assessed using fluorescence *in situ* hybridization (FISH) employing a human-specific genomic probe labeled as previously described [12]. Briefly, 3 mm sections were deparaffinized, washed with PBS for 5 min, digested with pepsin (0.1% in 0.1 M HCl) at 37 °C for 10 min, and dehydrated. Pretreated sections were incubated with a Cy3-labeled human-specific FISH probe (Chromosome Science Labo Inc., Sapporo, Japan), enclosed with coverslips, and simultaneously denatured at 90 °C for 10 min. Hybridization was conducted at 37 °C overnight. Sections were then washed with 50% formamide, 2× SSC at 37 °C for 20 min, and 1× SSC for 15 min at room temperature, followed by counterstaining with DAPI and mounting.

**Soft agar colony formation assay**

MEM (Thermo Fisher Scientific) supplemented with 10% FBS and Bacto Agar (BD Biosciences) solution was added to a 60 mm dish and allowed to solidify. hiPSC-CM patches were dissociated into single cells via treatment with 0.25% trypsin-EDTA solution (Thermo Fisher Scientific) and suspended in DMEM supplemented with 10% FBS. HeLa cells (JCRB Cell Bank, NIBIOHN, Osaka, Japan) were used as the positive control, while MRC-5 cells (DS Pharma Biomedical, Osaka, Japan) were used as the negative control. Cells were mixed with noble agar and spread onto a 60 mm dish precovered with a bottom layer. After placement onto the bottom agar layer, the top agar layers immediately solidified. The dishes were incubated with culture medium containing 10% FBS for 3 weeks at 37 °C and 5% CO_2_. At the end of the incubation period, colonies were visualized by staining with p-iodonitrotetrazolium violet (Nacalai Tesque) for 6 h at 37 °C and 5% CO_2._ Colony formation images were acquired using an inverted microscope (IX73; Olympus).

**General toxicity tests**

The hiPSC-CM patches were tested for general toxicity using immunodeficient NOD/Shi-scid, IL-2R γ^null^ mice (NOG mice; 7 weeks old, In-Vivo Science Inc., Tokyo, Japan) as described in Table S8. Seventy-six mice (38 male and 38 female) were obtained 7 days before the operation. The day after arrival, the mice were tagged for individual identification. Four to five mice were housed per cage. The cages were identified by labels showing the study number, cage number, strain, sex, number of animals, date of animal arrival, week's age at arrival, individual identification number, and the person responsible for the study. Mice were housed with bedding (autoclaved-wood shavings; Rettenmaier Japan Co., Ltd., Tokyo, Japan) and provided with water (tap water containing 2 ppm sodium hypochlorite) and food (Charles River Laboratories Japan, Inc., Kanagawa, Japan) *ad libitum*. The day before the operation, four larger and four smaller mice, male and female, respectively, were excluded from the groups and were considered spares. The remaining 60 mice were divided into 6 groups of 10 mice per group (3 groups for both males and females) by weight-stratified random sampling based on body weight. The mean +/- standard deviation of body weight (g) in each group is as follows: 23.3 +/- 0.6, 23.1 +/- 0.8, 23.4 +/- 0.7, 18.8 +/- 0.6, 18.7 +/- 1.0, and 18.9 +/- 0.8, n.s.). The mice groups were as follows: (1) nonoperated males; (2) sham-operated (open-chest) males; (3) hiPSC-CM-receiving males; (4) nonoperated females; (5) sham-operated females; and (6) hiPSC-CM-receiving females. For the hiPSC-CM-receiving group (groups 3 and 6), one hiPSC-CM patch consisting of 1.9 million hiPSC-CMs was directly transplanted onto the surface of the left anterior wall of the heart as well as the clinical application pathway. For the sham-operated groups (groups 2 and 5), the same procedure as the hiPSC-CM receiving groups was performed without hiPSC-CM patch transplantation. For the nonoperated groups (groups 1 and 4), surgery was not performed. The sample size was decided according to the ICH S4 harmonized tripartite guideline, duration of chronic toxicity testing in animals. If mice died immediately after surgery, they were replenished using mice from the spare cages. The treated number of mice in each group was as follows: 10 in group (1), 11 in group (2), 12 in group (3), 10 in group (4), 11 in group (5), and 10 in group (6). Mice that were excluded because of their weight (12 mice) or died immediately after surgery (4 mice) were excluded from the experiment. One mouse in group (2) died 2 days after surgery and was excluded from the analysis. Other than that instance, there was no observed abnormalities, and the mice gained weight steadily. There were no cases of mice during the study period that would be considered a humane endpoint with a poor prognosis. The exact number of mice used for analysis was as follows: 10 in group (1), 9 in group (2), 10 in group (3), 10 in group (4), 10 in group (5), and 10 in group (6). Twenty-eight days after transplantation, mice were exsanguinated under inhalation anesthesia with isoflurane (Mylan Inc., Canonsburg, PA) and dissected. Gross abnormalities and the weight of the major organs were recorded. Peripheral blood was collected to conduct hematological and biochemical evaluations. The analyses were performed by persons who were blinded to the grouping. For each sex, a test of equal variances (F test, two-tailed) was performed for body weight, hematological and biochemical parameters, and organ and tissue weights of untreated (Group (1) or (4)) and sham (Group (2) or (5)) and sham (Group (2) or (5)) and cell sheet transplanted (Group (3) or (6)) mice. If they F test was not significant (equal variance) significance testing was performed using Student's t test (two-tailed), and if the F test was significant (unequal variance), significance testing was performed using Aspin-Welch's t test (two-tailed). In all cases, the significance level was set at 5%. For histopathological examination, statistically significant difference tests were performed for each sex between no treatment (group (1) or (4)) and Sham (group (2) or (5)) and between Sham (group (2) or (5)) and cell sheet transplantation (group (3) or (6)) at a risk rate of 5% (P<0.05) or 1% (P<0.01). The results were determined. For testing differences in the frequency of occurrence of histopathological findings, Fisher's direct probability test (one-tailed) and Wilcoxon test (two-tailed) for lesions with degree were performed. Statistical testing was processed using the statistical analysis software EXSUS (Ver.8.1, EPS Corporation, Tokyo, Japan).

**Tumorigenicity assay**

The hiPSC-CM patch was tested for tumorigenicity using immunodeficient NOG mice (6-9 weeks old, female). As described above, an hiPSC-CM patch consisting of 1.9 million hiPSC-CMs, with or without purification and elimination of residual undifferentiated hiPSCs, was directly transplanted onto the surface of the left anterior wall of the heart. The mice were divided into 2 groups of 10 mice per group [13] by weight stratified random sampling based on body weight. Mouse survival and body weight following transplantation were recorded. Mice were euthanized under inhalation anesthesia with isoflurane and dissected 16 weeks after transplantation. The major organs and tissues were carefully observed, and any gross pathological findings were collected and stored for further examination.

**Whole-genome/whole-exome sequencing analysis**

We performed whole-genome sequencing (WGS), whole-exome sequencing (WES), and single nucleotide polymorphism (SNP) array experiments using peripheral blood mononuclear cells from the donor (control), the master cell bank (MCB), and the expansion cultures of hiPSC-CMs, and hiPSC-CM patches from the MCB.

We prepared 200 ng and 100 ng of genomic DNA as the starting material for WGS and WES, respectively. As per the manufacturer’s protocols, libraries for WGS were generated using the KAPA Hyper Prep Kit (Kapa Biosystems, Wilmington, MA, USA) without performing PCR on fragmented genomic DNA sheared by Covaris LE220 (Covaris, Brighton, UK). For WES, adapter-ligated libraries were prepared using the KAPA Hyper Prep Kit (Kapa Biosystems), and sequencing libraries were constructed using the SeqCap EZ Human Exome Library v3.0 (Roche, Basel, Switzerland). Cluster generation was performed with the HiSeq PE Cluster Kit v4 (Illumina) using Illumina cBot. Sequencing was performed using the HiSeq 2500 platform in the 126 paired-end mode. After the FASTQ files were generated (via bcl2fastq v2.17.1.14; Illumina) and adapter trimming was performed using cutadapt 1.10 [14], FASTQ files were mapped to the reference human genome (hg19 with decoy plasmid sequences for establishing hiPSCs and PhiX sequence) using BWA MEM (v0.7.15; with the default parameters, except for the use of the T-0 option for Genomon2) [15], and duplicated reads were removed using NovoSort (Novocraft; v1.03.09). The depths of coverage of WGS and WES data were 56×–83× and 84×–114× on average, respectively. To determine single nucleotide variants (SNVs) or insertions/deletions (indels) in the test samples (compared with the control sample), bam files were analyzed using the EB call function of Genomon (v1.0.1) [16] and Genomon2 (v2.3.0) [17]. The significance level, as per Fisher’s exact test used in Genomon, was *P* < 0.001 for WGS and WES. In Genomon2, *P* < 0.1 was used for WGS and WES, and the significance levels of the EB calls were set as *P* < 0.001 and *P* < 0.0001 for WGS and WES, respectively. After calling SNV and indels with Genomon and Genomon2, mutations whose variant allele frequencies were < 0.05 and fewer than five times those of the control sample were discarded. Thereafter, functional annotation was performed using ANNOVAR [18]; mutations were restricted to CDS and splicing sites. To further identify potentially pathogenic mutations, we excluded synonymous mutations and focused on mutations possibly related to cancer or other diseases. Mutations registered in the population databases SNP131 [19], esp6500si_all (> 0.01)[20], 1000g2014oct_all (> 0.01) [21], HGVD (v 2.0.0; > 0.01) [22], and 1KJPN (v1; > 0.01) [23] were discarded, but those registered in HGMD Pro (2016.4) [24], COSMIC79_position [25], COSMIC Cancer Gene Census (v79) [26], or Shibata’s list [27] were retained. Mutations that passed these filters were reported. WGS data were also used to determine copy number variations (CNVs) with VarScan2 (v2.4.2) [28] in combination with Otsu’s threshold method [29] and Delly [30] (v0.7.3) by comparing the test samples and the control sample. Finally, we manually curated candidate CNVs based on overall trends in the coverage depth, mapping status, and characteristics of the genomic regions, which were assessed by observing the positions of the candidate CNVs within the genome browser. We investigated genomic mutations by comparing the test and control samples.

**SNP array analysis**

CNVs were called using the HumanOmniExpress24 v1.1 genotyping array (Illumina). We prepared 200 ng of genomic DNA hybridized using the HumanOmniExpress24 v1.1 DNA Analysis Kit (Illumina) and evaluated its intensity using iScan (Illumina). After exporting a final report using GenomeStudio (v2011.1; Illumina), CNV analyses were performed using PennCNV (v1.0.3) [31], Mosaic Alteration Detection-MAD (v1.0.1) [32], and GWAS tools (v1.16.1) [33]; the test samples were compared with the control sample, and CNVs were manually curated.

**Supplemental Tables**

**Table S1. List of antibodies used in this study**

| Antibody | Vendor | Catalog No. |
| --- | --- | --- |
| cTNT | Abcam | ab45932 |
| cTNT | Thermo Fisher Scientific | MS-295-P |
| cTNT | Santa Cruz Biotechnology | sc-20025 |
| α-Actinin | Sigma-Aldrich | A7811 |
| α-Actinin | Abcam | ab68167 |
| Connexin43 | Sigma-Aldrich | C6219 |
| MLC2v | Proteintech | 10906-1-AP |
| MLC2a | Synaptic Systems | 311 011 |
| α-MHC | Sigma-Aldrich | HPA001349 |
| α-MHC | R&D | 940344 |
| β-MHC | Sigma-Aldrich | M8421 |
| LIN28A | LSBio | LS-B5073 |
| CD31 | Abcam | ab28364 |
| αSMA | Dako | M0851 |
| αSMA | Abcam | ab32575 |
| N-cadherin | Abcam | ab18203 |
| Vimentin | Abcam | ab92547 |
| Collagen I | Abcam | ab34710 |
| Laminin | Sigma-Aldrich | L9393 |
| Ki-67 | Dako | M7240 |
| Lamin | Abcam | ab108595 |
| Alexa Fluor 488 goat anti-mouse | Thermo Fisher Scientific | A11001 |
| Alexa Fluor 488 donkey anti-mouse | Thermo Fisher Scientific | A21202 |
| Alexa Fluor 555 donkey anti-mouse | Thermo Fisher Scientific | A31570 |
| Alexa Fluor 555 goat anti-rabbit | Thermo Fisher Scientific | A21428 |
| Alexa Fluor 488 donkey anti-rabbit | Thermo Fisher Scientific | A21206 |
| Alexa Fluor 555 donkey anti-rabbit | Thermo Fisher Scientific | A31572 |

cTNT, cardiac troponin T; MLC2a, atrial isoform of the myosin light chain 2; MLCv, ventricular isoform of myosin light chain; MHC, myosin heavy chain; SMA, smooth muscle actin.

**Table S2. List of primers used in this study**

| Gene Symbol | Primer | **Sequence** |
| --- | --- | --- |
| **Human** |  |  |
| *GAPDH* | SYBR | F:3′-CAATGACCCCTTCATTGACC-5′ R: 5′-TTGATTTTGGAGGGATCTCG-3′ |
| *POU5F1* | SYBR | F:3′-GAAACCCACACTGCAGCAGA-5′ R: 5′-TCGCTTGCCCTTCTGGCG-3′ |
| *SOX2* | SYBR | F:3′-GCGCCCTGCAGTACAACTC-5′ R: 5′-CGGACTTGACCACCGAACC-3′ |
| *NANOG* | SYBR | F:3′-CTCAGCTACAAACAGGTGAAGAC-5′ R: 5′-TCCCTGGTGGTAGGAAGAGTAAA-3′ |
| *Lin28A* | SYBR | F:3′-CACGGTGCGGGCATCTG-5′ R: 5′-CCTTCCATGTGCAGCTTACTC-3′ |
| *MESP1* | SYBR | F:3′-CAACTGACGCCGTCTCTGTGA-5′ R: 5′-GTCTGCCAAGGAACCACTTCG-3′ |
| *Brachyury* | SYBR | F:3′-AATTGGTCCAGCCTTGGAAT-5′ R: 5′-CGTTGCTCACAGACCACA-3′ |
| *EOMES* | SYBR | F:3′-CTTGCTAGGCCTCTGCTGTGTG-5′ R: 5′-TTGGTGACTCCTTAGCTTGCTCTCT-3′ |
| *Islet1* | SYBR | F:3′-TTTATTGTCGGAAGACTTGCCACTT-5′ R: 5′-TCAAAGACCACCGTACAACCTTTATCT-3′ |
| *PDGFRA* | SYBR | F:3′-TTGCTGTGAGCCTTGCATGA-5′ R: 5′-GTGGGAGCATTTGTTAGGACTGG-3′ |
| *MEF2C* | SYBR | F:3′-TCGCTTGTAAATGAGGGCATACAA-5′ R: 5′-GTCCAGCTTATGCCGCTGTG-3′ |
| *TNNT2* | SYBR | F:3′-GGCAGCTCCTGTTTGGAAATG-5′ R: 5′-TTATTACTGGTGTGGAGTGGGTGTG-3′ |
| *ACTN2* | SYBR | F:3′-TTTCCCTGTGTGTTGGTTGC-5′ R: 5′-TGATTACACTCCGCACATTTCA-3′ |
| *MYH6* | SYBR | F:3′-GAGATTTCTCCAACCCAG-5′ R: 5′-CCAGGGTGATGGAGAAGGAG-3′ |
| *MYH7* | SYBR | F:3′-TTTCCCTGTGTGTTGGTTGC-5′ R: 5′-TGATTACACTCCGCACATTTCA-3′ |
| *KCNQ1* | SYBR | F:3′-GAGATTTCTCCAACCCAG-5′ R: 5′-CCAGGGTGATGGAGAAGGAG-3′ |
| *KCNH2* | SYBR | F:3′-TTTCCCTGTGTGTTGGTTGC-5′ R: 5′-TGATTACACTCCGCACATTTCA-3′ |
| *CACNA1C* | SYBR | F:3′-GAGATTTCTCCAACCCAG-5′ R: 5′-CCAGGGTGATGGAGAAGGAG-3′ |
| *SCN5A* | SYBR | F:3′-TTTCCCTGTGTGTTGGTTGC-5′ R: 5′-TGATTACACTCCGCACATTTCA-3′ |
| *SERCA2* | SYBR | F:3′-GAGATTTCTCCAACCCAG-5′ R: 5′-CCAGGGTGATGGAGAAGGAG-3′ |
| **Porcine** |  |  |
| *GAPDH* | TaqMan | Ss03374854_g1 |
| *SDF-1* | TaqMan | Ss03391855_m1 |
| *VEGF* | TaqMan | Ss03393993_m1 |
| *basic FGF* | TaqMan | Ss03375809_u1 |
| *HGF* | TaqMan | AJVI4PJ |

F, forward; R, reverse

# Table S3. List of plasmids used for hiPSC reprogramming

| Plasmid | Gene | Reference |
| --- | --- | --- |
| pCE-hSK | *SOX2, KLF4* | [34] |
| pCE-hUL | *L-MYC, LIN28* | [34] |
| pCE-hOCT3/4 | *OCT3/4* | [34] |
| pCE-mp53DD | *Trp53* | [34] |
| pCXB-EBNA1 | *EBNA1* | [34] |

**Table S4. Characterization of hiPSCs**

| **Assay** | **Method** | **Criteria** | **Results** |
| --- | --- | --- | --- |
| Morphology | Microscopic examination | Human ES cell-like | Human ES cell-like |
| Remaining plasmid vector | qPCR | Not detected | Not detected |
| Karyotype analysis | Conventional Giemsa  G-band | Normal (22 pairs of autosomal chromosomes and one pair of sex chromosomes) | Normal (22 pairs of autosomal chromosomes and one pair of sex chromosomes) |
| Expression of pluripotent markers | RNA microarray | *POU5F1*: ≥ 4%, *NANOG*: ≥ 5%, vs. *GAPDH* | Within the standard values |
|  | Flow cytometry | *SSEA4*: ≥ 90%, *TRA-1-60*: ≥ 90%, *TRA-2-49*: ≥ 90% | Within the standard values |
| Expression of differentiation resistance markers | RNA microarray | *C4orf51:* ≤ 0.04%, *ABHD12B:* ≤ 0.07%, *HHLA1:* ≤ 0.25% vs*.* *GAPDH* | Within the standard values |
| Doubling time | Calculated from cell growth curve | 15–45 h | Within the standard values |
| Sterility testing | BacT/ALERT ® MB | Negative | Negative |
| Mycoplasma testing | PCR | Negative | Negative |
| Endotoxin testing | Kinetic turbidimetric technique | ≤ 5 EU/mL | Within the criterion |
| Viral testing | PCR (*HBC, HCV, HIV, HTLV*, and *Parvovirus 19*) | All negative | All negative |
| HLA typing | PCR-SBT (one of each from *HLA-A, HLA-B*, and *HLA-DR*) | Match with the donor blood cell profile | Match with the donor cell profile |
| STR genotyping | PCR-capillary electrophoresis | Match with the donor blood cell profile | Match with the donor cell profile |

ES, embryonic stem cell; qPCR, quantitative PCR.

**Table S5. Quality tests for master cell bank (MCB)**

| **Assay** | **Method** | **Criteria** | **Results** |
| --- | --- | --- | --- |
| Sterility test | Direct inoculation method | Negative | Negative |
| Endotoxin test | Kinetic turbidimetric test | Negative | Negative |
| Mycoplasma test | DNA staining method and direct culture method | Negative | Negative |
| 200 Median cell profiles | Examination via transmission electron microscopy to detect viruses; virus-like particles; or extraneous agents, including mycoplasma, yeast, fungi, or bacteria | Negative | Negative |
| Detection of reverse transcriptase enzymatic activity assay | Real-time fluorescent product enhanced reverse transcriptase (F-PERT) assay | Negative | Negative |
| Detection of human viral pathogens | Real-Time PCR (*HIV1* and *2 provirus*, *HAV*, *HBV, HCV, HHV-6, HHV-7, HHV-8, hCMV, EBV, SV40,* and *B19*) | Negative | Negative |
| *In vitro* virus assay | Inoculated into MRC-5, Vero C1008, and HeLa cell cultures and assessed for the presence of cytopathology or hemadsorbing virus contamination | Negative | Negative |
| *In vivo* virus assay | Inoculated into adult mice, suckling mice, guinea pigs, and embryonated eggs, and assessed for any adventitious agents | Negative | Negative |
| Retrovirus assays | Co-cultivation assay using F-PERT | Negative | Negative |
| STR genotyping | PCR-capillary electrophoresis | Match with the original iPS cell profile | Match with the original iPS cell profile |

**Table S6. Quality test of hiPSC-CMs**

| **Assay** | **Method** | **Criteria** | **Results** |
| --- | --- | --- | --- |
| Viability | Trypan Blue exclusion test | ≥ 40% | 62.1% |
| Purity of cardiomyocytes | Flow cytometry | cTNT-positive rate ≥ 50% | 77.3% |
| Sterility testing | Membrane filtration method | Negative | Negative |
| Mycoplasma testing | Nested PCR | Negative | Negative |
| Endotoxin testing | Turbidimetric technique | < 1.0 EU/mL | < 0.194 EU/mL |

hiPSC-CM, human induced pluripotent stem cell-derived cardiomyocyte; cTNT, cardiac troponin T

**Table S7. Representative results of telemetered Holter electrocardiography**

| ID | Group | Pre-operation | After transplantation | | | | | | | |
| --- | --- | --- | --- | --- | --- | --- | --- | --- | --- | --- |
|  |  |  | 0–72 h | 1 w | 2 w | 4 w | 6 w | 8 w | 10 w | 12 w |
| 1 | Sham | 0 | 0 | 0 | 0 | 0 | 0 | 0 | 0 | 0 |
| 2 | Sham | 0 | 0 | 0 | 0 | 0 | 0 | 0 | 0 | 0 |
| 3 | Sham | 0 | 0 | 0 | 0 | 0 | 0 | 0 | 0 | 0 |
| 4 | Sham | 0 | 0 | 0 | 0 | 0 | 0 | 0 | 0 | 0 |
| 5 | hiPSC-CMs | 0 | 0 | 0 | 0 | 0 | 0 | 0 | 0 | 0 |
| 6 | hiPSC-CMs | 0 | 0 | 0 | 0 | 0 | 0 | 0 | 0 | 0 |
| 7 | hiPSC-CMs | 0 | 0 | 0 | 0 | 0 | 0 | 0 | 0 | 0 |
| 8 | hiPSC-CMs | 0 | 0 | 0 | 0 | 0 | 0 | 0 | 0 | 0 |
| 9 | hiPSC-CMs | 0 | 0 | 0 | 0 | 0 | 0 | 0 | 0 | 0 |
| 10 | hiPSC-CMs | 0 | 0 | 0 | 0 | 0 | 0 | 0 | 0 | 0 |
| 11 | hiPSC-CMs | 0 | 0 | 0 | 0 | 0 | 0 | 0 | 0 | 0 |

w, weeks.

**Table S8. Summary of the general toxicity and tumorigenicity tests**

|  |  | General toxicity | Tumorigenicity |
| --- | --- | --- | --- |
| Recipient | Strain | NOD/Shi-scid, IL2RγKO Jic mouse | |
|  | Age | 6–9 weeks old | |
|  | Sex | Male, female | Female |
|  | Group | 1. hiPSC-CM patch-receiving 2. Sham-operated 3. Non-operated | hiPSC-CM patch-receiving |
|  | Number | n = 10 | n = 10 |
| Transplant | | 1.9 × 10^6^ hiPSC-CMs per patch, 1 sheet per mouse | |
| Follow-up | | 28 days | 16 weeks |
| Assessment | Hematology | RBC, HGB, HCT, MCV, MCH, MCHC, PLT, WBC, Neut, Lympho, Mono, EO, and BASO | None |
|  | Biochemistry | ALP, AST, ALT, TCHO, TG, BUN, and CRE | None |
|  | Necropsy | Brain, spinal cord, pituitary, eyes, Harderian glands, tongue, salivary glands, thyroid glands, trachea, heart, lung, esophagus, aorta, liver, gallbladder, stomach, small intestine, large intestine, spleen, pancreas, kidneys, adrenal glands, urinary bladder, sciatic nerve, muscle, skin, sternum, femur, ovary, uterus, vagina, testis*, epididymis*, vesicular glands*, prostate glands*, and cervical lymph node | |
|  | Wet weight | Brain, liver, spleen, kidneys, adrenal glands, ovary, uterus, testis*, vesicular glands*, and prostate* | Brain, liver, spleen, kidneys, pituitary, heart, and lung |
|  | Pathology | H&E staining | H&E staining and immunohistochemistry as needed (anti-laminin, anti-Ki-67) |

*male mice only. HGB, hemoglobin; HCT, hematocrit; MCV, mean corpuscular volume; MCH, mean corpuscular hemoglobin; MCHC, mean corpuscular hemoglobin concentration; PLT, platelet; Neut, neutrophils; Lympho, lymphocytes; Mono, monocytes; EO, eosinophil; BASO, basophil; ALP, alkaline phosphatase; AST, aspartate aminotransferase; ALT, alanine aminotransferase; TCHO, total cholesterol; TG, triglyceride; BUN, blood urea nitrogen; CRE, creatinine.

**Table S9. Change of body weight in the general toxicity test**

| Group |  | Days after operation | | | | |
| --- | --- | --- | --- | --- | --- | --- |
|  |  | 0 | 7 | 14 | 21 | 28 |
| 1 | Average | 23.3 | 23.9 | 24.8 | 25.9 | 26.8 |
|  | SD | 0.6 | 1.2 | 1.3 | 1.2 | 1.2 |
| 2 | Average | 23.1 | 23.1 | 24.3 | 25.2 | 26.2 |
|  | SD | 0.8 | 0.9 | 1.3 | 1.2 | 1.3 |
|  | *t* test (v.s. group 1) | n.s. | n.s. | n.s. | n.s. | n.s. |
| 3 | Average | 23.4 | 23.4 | 24.7 | 25.4 | 26.0 |
|  | SD | 0.7 | 0.8 | 1.1 | 0.9 | 0.9 |
|  | *t*-test (v.s. group 2) | n.s. | n.s. | n.s. | n.s. | n.s. |
| 4 | Average | 18.8 | 19.0 | 19.9 | 20.4 | 21.4 |
|  | SD | 0.6 | 0.7 | 0.4 | 0.6 | 1.0 |
| 5 | Average | 18.7 | 18.4 | 19.8 | 20.5 | 21.5 |
|  | SD | 1.0 | 0.5 | 0.5 | 0.9 | 1.0 |
|  | *t*-test (v.s. group 4) | n.s. | *P* < 0.05 | n.s. | n.s. | n.s. |
| 6 | Average | 18.9 | 18.7 | 20.5 | 20.9 | 21.9 |
|  | SD | 0.8 | 1.0 | 0.9 | 0.8 | 0.9 |
|  | *t*-test (v.s. group 5) | n.s. | n.s. | n.s. | n.s. | n.s. |

n.s.: not significant

**Table S10. Hematology in the general toxicity test**

| Group | | RBC | | HGB | | HCT | | | MCV | | MCH | | MCHC | | PLT | | WBC | Neut | | Lympho | | Mono | | EO | | BASO |
| --- | --- | --- | --- | --- | --- | --- | --- | --- | --- | --- | --- | --- | --- | --- | --- | --- | --- | --- | --- | --- | --- | --- | --- | --- | --- | --- |
|  |  | × 10^4^ cells/μL | | g/dL | | % | | | fL | | pg | | g/dL | | × 10^4^ cells / μL | | × 10^2^ cells/μL | × 10^2^ cells/μL | | × 10^2^ cells/μL | | × 10^2^ cells/μL | | × 10^2^ cells/μL | | × 10^2^ cells/μL |
| 1 (n = 10) | Average | 816 | | 12.8 | | 40.7 | | | 49.9 | | 15.6 | | 31.4 | | 130.7 | | 4.6 | 3.5 | | 0.6 | | 0.4 | | 0.1 | | 0.0 |
|  | SD | 16 | | 0.3 | | 0.8 | | | 0.6 | | 0.2 | | 0.4 | | 4.8 | | 1.2 | 0.9 | | 0.3 | | 0.2 | | 0.0 | | 0.0 |
| 2 (n = 9) | Average | 830 | | 12.9 | | 41.7 | | | 50.2 | | 15.6 | | 31.0 | | 132.1 | | 5.6 | 4.3 | | 0.6 | | 0.7 | | 0.1 | | 0.0 |
|  | SD | 25 | | 0.4 | | 0.6 | | | 1.1 | | 0.1 | | 0.6 | | 6.1 | | 1.3 | 1.3 | | 0.3 | | 0.2 | | 0.0 | | 0.0 |
|  | *t*-test (v.s. group 1) | n.s. | | n.s. | | *P* < 0.01 | | | n.s. | | n.s. | | n.s. | | n.s. | | n.s. | n.s. | | n.s. | | *P* < 0.01 | | n.s. | | n.d. |
| 3 (n = 10) | Average | 842 | | 13.1 | | 41.6 | | | 49.4 | | 15.5 | | 31.4 | | 145.8 | | 5.0 | 3.6 | | 0.8 | | 0.6 | | 0.1 | | 0.0 |
|  | SD | 22 | | 0.4 | | 0.8 | | | 0.6 | | 0.1 | | 0.4 | | 9.9 | | 1.3 | 1.0 | | 0.3 | | 0.2 | | 0.0 | | 0.0 |
|  | *t*-test (v.s. group 2) | n.s. | | n.s. | | n.s. | | | n.s. | | n.s. | | n.s. | | *P* < 0.01 | | n.s. | n.s. | | n.s. | | n.s. | | n.s. | | n.d. |
| 4 (n = 10) | Average | 854 | | 13.6 | | 42.7 | | | 50.0 | | 15.9 | | 31.9 | | 112.8 | | 6.1 | 4.8 | | 0.6 | | 0.5 | | 0.1 | | 0.0 |
|  | SD | 24 | | 0.4 | | 1.0 | | | 0.7 | | 0.1 | | 0.4 | | 11.8 | | 2.1 | 1.7 | | 0.3 | | 0.3 | | 0.2 | | 0.0 |
| 5 (n = 10) | Average | 844 | | 13.5 | | 42.2 | | | 50.0 | | 16.0 | | 32.0 | | 126.4 | | 4.8 | 3.7 | | 0.5 | | 0.5 | | 0.1 | | 0.0 |
|  | SD | 24 | | 0.4 | | 1.3 | | | 0.9 | | 0.2 | | 0.4 | | 13.1 | | 1.9 | 1.7 | | 0.3 | | 0.2 | | 0.1 | | 0.0 |
|  | *t*-test (v.s. group 4) | n.s. | | n.s. | | n.s. | | | n.s. | | n.s. | | n.s. | | *P* < 0.05 | | n.s. | n.s. | | n.s. | | n.s. | | n.s. | | n.d. |
| 6 (n = 10) | Average | 841 | | 13.4 | | 41.8 | | | 49.7 | | 15.9 | | 32.0 | | 132.1 | | 4.6 | 3.8 | | 0.4 | | 0.3 | | 0.0 | | 0.0 |
|  | SD | 18 | 0.3 | | 0.5 | | 1.0 | 0.2 | | 0.5 | | 11.2 | | 1.5 | | 1.4 | | | 0.3 | | 0.2 | | 0.1 | | 0.0 | |
|  | *t*-test (v.s. group 5) | n.s. | n.s. | | n.s. | | n.s. | n.s. | | n.s. | | n.s. | | n.s. | | n.s. | | | n.s. | | *P* < 0.05 | | n.s. | | n.d. | |

n.s.: not significant, n.d.: not detectable

**Table S11. Biochemistry results in the general toxicity test**

| Group | | ALP | AST | ALT | TCHO | TG | BUN | CRE |
| --- | --- | --- | --- | --- | --- | --- | --- | --- |
|  |  | U/L | U/L | U/L | mg/dL | mg/dL | mg/dL | mg/dL |
| 1 (n = 10) | Average | 171 | 58 | 26 | 70 | 126 | 29.3 | 0.2 |
|  | SD | 22 | 21 | 7 | 8 | 66 | 2.1 | 0.1 |
| 2 (n = 9) | Average | 166 | 49 | 23 | 77 | 86 | 23.6 | 0.1 |
|  | SD | 22 | 14 | 5 | 7 | 20 | 2.4 | 0.1 |
|  | *t*-test (v.s. group 1) | n.s. | n.s. | n.s. | n.s. | n.s. | *P* < 0.001 | n.s. |
| 3 (n = 10) | Average | 182 | 43 | 20 | 74 | 115 | 24.5 | 0.1 |
|  | SD | 26 | 5 | 3 | 4 | 45 | 2.1 | 0.0 |
|  | *t-*test (v.s. group 2) | n.s. | n.s. | n.s. | n.s. | n.s. | n.s. | n.s. |
| 4 (n = 10) | Average | 235 | 41 | 18 | 64 | 79 | 20.0 | 0.1 |
|  | SD | 38 | 2 | 3 | 5 | 30 | 1.8 | 0.0 |
| 5 (n = 10) | Average | 242 | 42 | 18 | 62 | 77 | 21.4 | 0.1 |
|  | SD | 31 | 8 | 3 | 8 | 31 | 2.7 | 0.0 |
|  | *t*-test (v.s. group 4) | n.s. | n.s. | n.s. | n.s. | n.s. | n.s. | n.s. |
| 6 (n = 10) | Average | 242 | 39 | 17 | 61 | 85 | 21.5 | 0.1 |
|  | SD | 31 | 5 | 2 | 5 | 22 | 2.8 | 0.0 |
|  | t-test (v.s. group 5) | n.s. | n.s. | n.s. | n.s. | n.s. | n.s. | n.s. |

n.s.: not significant

**Table S12. Organs and tissue weight in the general toxicity test**

| Group | | Brain | Liver | Spleen | Kidney | | Adrenal gland | | Testis | | Vesicular gland | Prostate gland | Uterus | Ovary | |
| --- | --- | --- | --- | --- | --- | --- | --- | --- | --- | --- | --- | --- | --- | --- | --- |
|  |  |  |  |  | right | left | right | left | right | left |  |  |  | right | left |
| 1 (n = 10) | Average | 0.506 | 1.370 | 0.024 | 0.202 | 0.191 | 0.005 | 0.005 | 0.097 | 0.091 | 0.293 | 0.063 |  |  |  |
|  | SD | 0.019 | 0.090 | 0.002 | 0.011 | 0.015 | 0.002 | 0.002 | 0.012 | 0.013 | 0.035 | 0.020 |  |  |  |
| 2 (n = 9) | Average | 0.494 | 1.371 | 0.028 | 0.200 | 0.193 | 0.005 | 0.005 | 0.101 | 0.102 | 0.268 | 0.042 |  |  |  |
|  | SD | 0.023 | 0.098 | 0.003 | 0.015 | 0.008 | 0.001 | 0.001 | 0.008 | 0.014 | 0.035 | 0.012 |  |  |  |
|  | *t*-test (v.s. group 1) | n.s. | n.s. | *P* < 0.01 | n.s. | n.s. | n.s. | n.s. | n.s. | n.s. | n.s. | n.s. |  |  |  |
| 3 (n = 10) | Average | 0.502 | 1.287 | 0.025 | 0.195 | 0.184 | 0.005 | 0.005 | 0.100 | 0.094 | 0.292 | 0.055 |  |  |  |
|  | SD | 0.011 | 0.088 | 0.002 | 0.015 | 0.011 | 0.001 | 0.002 | 0.006 | 0.006 | 0.030 | 0.014 |  |  |  |
|  | *t*-test (v.s. group 2) | n.s. | n.s. | *P* < 0.05 | n.s. | *P* < 0.05 | n.s. | n.s. | n.s. | n.s. | n.s. | n.s. |  |  |  |
| 4 (n = 10) | Average | 0.503 | 0.954 | 0.031 | 0.128 | 0.119 | 0.004 | 0.005 |  |  |  |  | 0.011 | 0.011 | 0.147 |
|  | SD | 0.020 | 0.060 | 0.006 | 0.009 | 0.006 | 0.001 | 0.001 |  |  |  |  | 0.002 | 0.002 | 0.048 |
| 5 (n = 10) | Average | 0.504 | 0.949 | 0.028 | 0.127 | 0.119 | 0.004 | 0.005 |  |  |  |  | 0.010 | 0.012 | 0.131 |
|  | SD | 0.015 | 0.061 | 0.006 | 0.009 | 0.006 | 0.001 | 0.001 |  |  |  |  | 0.002 | 0.003 | 0.058 |
|  | *t*-test (v.s. group 4) | n.s. | n.s. | n.s. | n.s. | n.s. | n.s. | n.s. |  |  |  |  | n.s. | n.s. | n.s. |
| 6 (n = 10) | Average | 0.506 | 0.955 | 0.031 | 0.134 | 0.121 | 0.005 | 0.005 |  |  |  |  | 0.010 | 0.010 | 0.109 |
|  | SD | 0.021 | 0.072 | 0.005 | 0.012 | 0.004 | 0.001 | 0.001 |  |  |  |  | 0.002 | 0.002 | 0.027 |
|  | *t*-test (v.s. group 5) | n.s. | n.s. | n.s. | n.s. | n.s. | n.s. | n.s. |  |  |  |  | n.s. | n.s. | n.s. |

n.s.: not significant

**Table S13. Pathological findings in the general toxicity test**

| Organ and findings | Sex | Male | | | | | |  | Female | | | | | |
| --- | --- | --- | --- | --- | --- | --- | --- | --- | --- | --- | --- | --- | --- | --- |
|  | Group | 1 | | 2 | | 3 | |  | 4 | | 5 | | 6 | |
| No. of animals /group | | 10 |  | 9 |  | 10 |  |  | 10 |  | 10 |  | 10 |  |
| Heart |  |  |  |  |  |  |  |  |  |  |  |  |  |  |
|  | Normal | 10 |  | 4 |  | 1 |  |  | 10 |  | 2 |  | 0 |  |
|  | Fibrosis, pericardium (minimal) | 0 |  | 5 | *P* < 0.01 vs. group 1 | 8 |  |  | 0 |  | 5 | *P* < 0.01 vs. group 4 | 4 |  |
|  | Fibrosis, pericardium (slight) | 0 |  | 0 |  | 1 |  |  | 0 |  | 3 |  | 6 |  |
| Aorta |  |  |  |  |  |  |  |  |  |  |  |  |  |  |
|  | Normal | 10 |  | 9 |  | 10 |  |  | 10 |  | 10 |  | 10 |  |
| Mandibular lymph node |  |  |  |  |  |  |  |  |  |  |  |  |  |  |
|  | Not examined | 1 |  | 2 |  | 2 |  |  | 3 |  | 2 |  | 3 |  |
|  | Lymphoid depletion (severe) | 9 |  | 7 |  | 8 |  |  | 7 |  | 8 |  | 7 |  |
| Spleen |  |  |  |  |  |  |  |  |  |  |  |  |  |  |
|  | Extramedullary hematopoiesis (minimal) | 0 |  | 1 |  | 0 |  |  | 0 |  | 3 |  | 3 |  |
|  | Lacks lymphoid follicles (severe) | 10 |  | 9 |  | 10 |  |  | 10 |  | 10 |  | 10 |  |
|  | Osseous metaplasia (slight) | 0 |  | 1 |  | 0 |  |  | 0 |  | 0 |  | 0 |  |
| Bone marrow |  |  |  |  |  |  |  |  |  |  |  |  |  |  |
|  | Normal | 10 |  | 9 |  | 10 |  |  | 10 |  | 10 |  | 10 |  |
| Pituitary |  |  |  |  |  |  |  |  |  |  |  |  |  |  |
|  | Normal | 10 |  | 9 |  | 10 |  |  | 10 |  | 10 |  | 10 |  |
| Thyroid |  |  |  |  |  |  |  |  |  |  |  |  |  |  |
|  | Normal | 10 |  | 9 |  | 10 |  |  | 10 |  | 10 |  | 10 |  |
| Adrenal |  |  |  |  |  |  |  |  |  |  |  |  |  |  |
|  | Normal | 10 |  | 7 |  | 9 |  |  | 0 |  | 1 |  | 1 |  |
|  | Hyperplasia, subcapsular cell | 0 |  | 2 |  | 1 |  |  | 10 |  | 9 |  | 9 |  |
| Trachea |  |  |  |  |  |  |  |  |  |  |  |  |  |  |
|  | Normal | 10 |  | 9 |  | 10 |  |  | 10 |  | 10 |  | 10 |  |
| Lung/bronchial |  |  |  |  |  |  |  |  |  |  |  |  |  |  |
|  | Normal | 10 |  | 9 |  | 10 |  |  | 10 |  | 10 |  | 10 |  |
| Tongue |  |  |  |  |  |  |  |  |  |  |  |  |  |  |
|  | Normal | 10 |  | 9 |  | 10 |  |  | 10 |  | 10 |  | 10 |  |
| Salivary gland |  |  |  |  |  |  |  |  |  |  |  |  |  |  |
|  | Normal | 10 |  | 9 |  | 10 |  |  | 10 |  | 10 |  | 10 |  |
| Esophagus |  |  |  |  |  |  |  |  |  |  |  |  |  |  |
|  | Normal | 10 |  | 9 |  | 10 |  |  | 10 |  | 10 |  | 10 |  |
| Stomach |  |  |  |  |  |  |  |  |  |  |  |  |  |  |
|  | Normal | 9 |  | 9 |  | 10 |  |  | 10 |  | 10 |  | 10 |  |
|  | Cyst, glandular (minimal) | 0 |  | 0 |  | 0 |  |  | 0 |  | 0 |  | 0 |  |
|  | Epidermal cyst (minimal) | 1 |  | 0 |  | 0 |  |  | 0 |  | 0 |  | 0 |  |
| Duodenum |  |  |  |  |  |  |  |  |  |  |  |  |  |  |
|  | Normal | 10 |  | 9 |  | 10 |  |  | 10 |  | 10 |  | 10 |  |
| Jejunum |  |  |  |  |  |  |  |  |  |  |  |  |  |  |
|  | Normal | 10 |  | 9 |  | 10 |  |  | 10 |  | 10 |  | 10 |  |
| Ileum |  |  |  |  |  |  |  |  |  |  |  |  |  |  |
|  | Normal | 10 |  | 9 |  | 10 |  |  | 10 |  | 10 |  | 10 |  |
| Cecum |  |  |  |  |  |  |  |  |  |  |  |  |  |  |
|  | Normal | 10 |  | 9 |  | 10 |  |  | 10 |  | 10 |  | 10 |  |
| Colon |  |  |  |  |  |  |  |  |  |  |  |  |  |  |
|  | Normal | 10 |  | 9 |  | 10 |  |  | 10 |  | 10 |  | 10 |  |
| Rectum |  |  |  |  |  |  |  |  |  |  |  |  |  |  |
|  | Normal | 10 |  | 9 |  | 10 |  |  | 10 |  | 10 |  | 10 |  |
| Pancreas |  |  |  |  |  |  |  |  |  |  |  |  |  |  |
|  | Normal | 10 |  | 8 |  | 10 |  |  | 10 |  | 10 |  | 9 |  |
|  | Infiltrate, mixed cell (minimal) | 0 |  | 0 |  | 0 |  |  | 0 |  | 0 |  | 1 |  |
|  | Necrosis (slight) | 0 |  | 1 |  | 0 |  |  | 0 |  | 0 |  | 0 |  |
| Liver |  |  |  |  |  |  |  |  |  |  |  |  |  |  |
|  | Normal | 10 |  | 9 |  | 10 |  |  | 10 |  | 10 |  | 10 |  |
| Gall bladder |  |  |  |  |  |  |  |  |  |  |  |  |  |  |
|  | Normal | 10 |  | 9 |  | 10 |  |  | 10 |  | 10 |  | 10 |  |
| Kidney |  |  |  |  |  |  |  |  |  |  |  |  |  |  |
|  | Normal | 10 |  | 9 |  | 10 |  |  | 10 |  | 10 |  | 10 |  |
| Urinary bladder |  |  |  |  |  |  |  |  |  |  |  |  |  |  |
|  | Normal | 10 |  | 9 |  | 10 |  |  | 10 |  | 10 |  | 10 |  |
| Testis |  |  |  |  |  |  |  |  |  |  |  |  |  |  |
|  | Normal | 10 |  | 8 |  | 10 |  |  |  |  |  |  |  |  |
|  | Dilatation, tubular (slight) | 0 |  | 1 |  | 0 |  |  |  |  |  |  |  |  |
| Prostate |  |  |  |  |  |  |  |  |  |  |  |  |  |  |
|  | Normal | 10 |  | 9 |  | 10 |  |  |  |  |  |  |  |  |
| Epididymis |  |  |  |  |  |  |  |  |  |  |  |  |  |  |
|  | Normal | 10 |  | 8 |  | 9 |  |  |  |  |  |  |  |  |
|  | Sperm granuloma (minimal) | 0 |  | 1 |  | 0 |  |  |  |  |  |  |  |  |
|  | Sperm granuloma (slight) | 0 |  | 0 |  | 1 |  |  |  |  |  |  |  |  |
| Seminal vesicle |  |  |  |  |  |  |  |  |  |  |  |  |  |  |
|  | Normal | 10 |  | 9 |  | 10 |  |  |  |  |  |  |  |  |
| Mammary gland |  |  |  |  |  |  |  |  |  |  |  |  |  |  |
|  | Normal | 0 |  | 0 |  | 0 |  |  | 10 |  | 10 |  | 10 |  |
|  | Not examined | 10 |  | 9 |  | 10 |  |  | 0 |  | 0 |  | 0 |  |
| Ovary |  |  |  |  |  |  |  |  |  |  |  |  |  |  |
|  | Normal |  |  |  |  |  |  |  | 10 |  | 10 |  | 10 |  |
| Uterus |  |  |  |  |  |  |  |  |  |  |  |  |  |  |
|  | Normal |  |  |  |  |  |  |  | 10 |  | 9 |  | 10 |  |
|  | Dilatation, lumen (slight) |  |  |  |  |  |  |  | 0 |  | 1 |  | 0 |  |
| Vagina |  |  |  |  |  |  |  |  |  |  |  |  |  |  |
|  | Normal |  |  |  |  |  |  |  | 10 |  | 10 |  | 10 |  |
| Femur |  |  |  |  |  |  |  |  |  |  |  |  |  |  |
|  | Normal | 10 |  | 9 |  | 10 |  |  | 10 |  | 10 |  | 10 |  |
| Sternum |  |  |  |  |  |  |  |  |  |  |  |  |  |  |
|  | Normal | 10 |  | 9 |  | 10 |  |  | 10 |  | 10 |  | 10 |  |
| Musculature |  |  |  |  |  |  |  |  |  |  |  |  |  |  |
|  | Normal | 10 |  | 9 |  | 10 |  |  | 10 |  | 10 |  | 10 |  |
| Skin/subcutis |  |  |  |  |  |  |  |  |  |  |  |  |  |  |
|  | Normal | 10 |  | 9 |  | 10 |  |  | 10 |  | 10 |  | 10 |  |
| Eye |  |  |  |  |  |  |  |  |  |  |  |  |  |  |
|  | Normal | 9 |  | 8 |  | 10 |  |  | 6 |  | 8 |  | 8 |  |
|  | Cataract (minimal) | 1 |  | 1 |  | 0 |  |  | 4 |  | 0 |  | 1 |  |
|  | Cataract (slight) | 0 |  | 0 |  | 0 |  |  | 0 |  | 0 |  | 1 |  |
|  | Cataract (moderate) | 0 |  | 0 |  | 0 |  |  | 0 |  | 2 |  | 0 |  |
|  | Corneal atrophy (moderate) | 0 |  | 0 |  | 0 |  |  | 0 |  | 2 |  | 0 |  |
| Harderian gland |  |  |  |  |  |  |  |  |  |  |  |  |  |  |
|  | Normal | 10 |  | 8 |  | 10 |  |  | 10 |  | 10 |  | 10 |  |
|  | Hemorrhage (minimal) | 0 |  | 1 |  | 0 |  |  | 0 |  | 0 |  | 0 |  |
| Brain |  |  |  |  |  |  |  |  |  |  |  |  |  |  |
|  | Normal | 10 |  | 8 |  | 10 |  |  | 10 |  | 10 |  | 10 |  |
|  | Hematoma, lipomatous (slight) | 0 |  | 1 |  | 0 |  |  | 0 |  | 0 |  | 0 |  |
| Spinal cord |  |  |  |  |  |  |  |  |  |  |  |  |  |  |
|  | Normal | 10 |  | 9 |  | 10 |  |  | 10 |  | 10 |  | 10 |  |
| Sciatic nerve |  |  |  |  |  |  |  |  |  |  |  |  |  |  |
|  | Normal | 10 |  | 9 |  | 10 |  |  | 10 |  | 10 |  | 10 |  |

**Supplemental Figures**

**
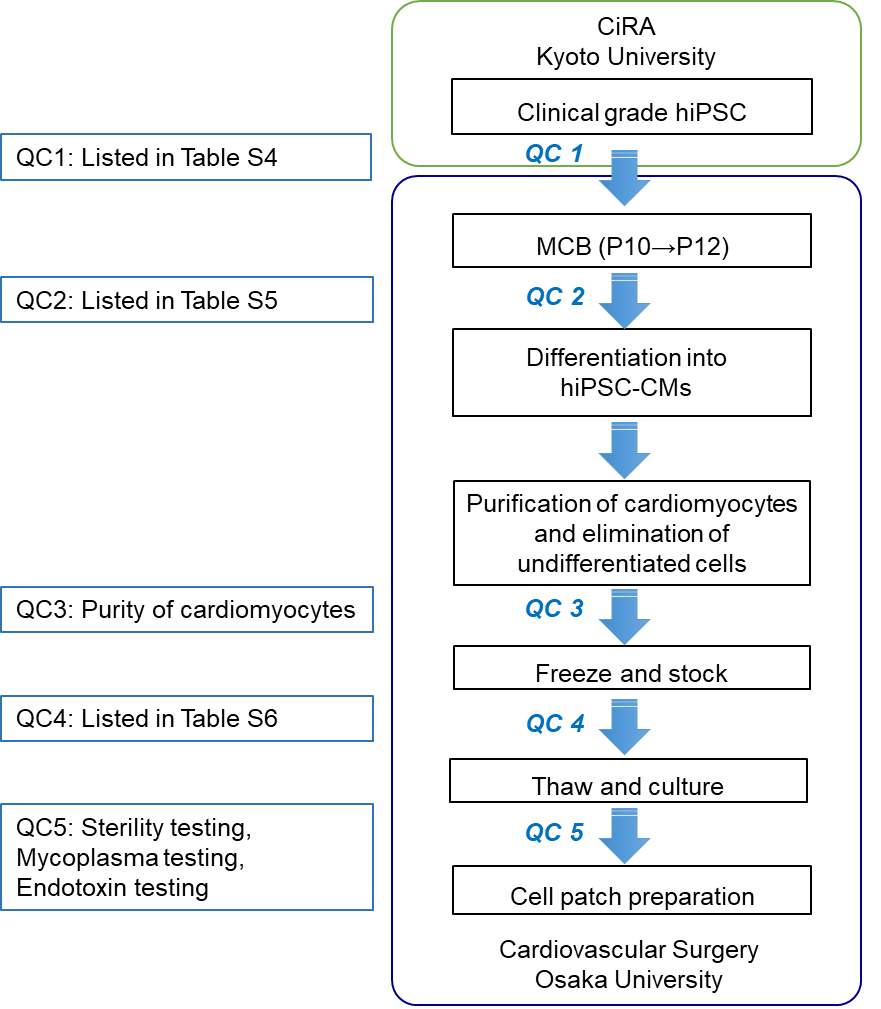
**

**Figure S1. Quality checks of hiPSCs, hiPSC-CMs, and the hiPSC-CM patch**

**
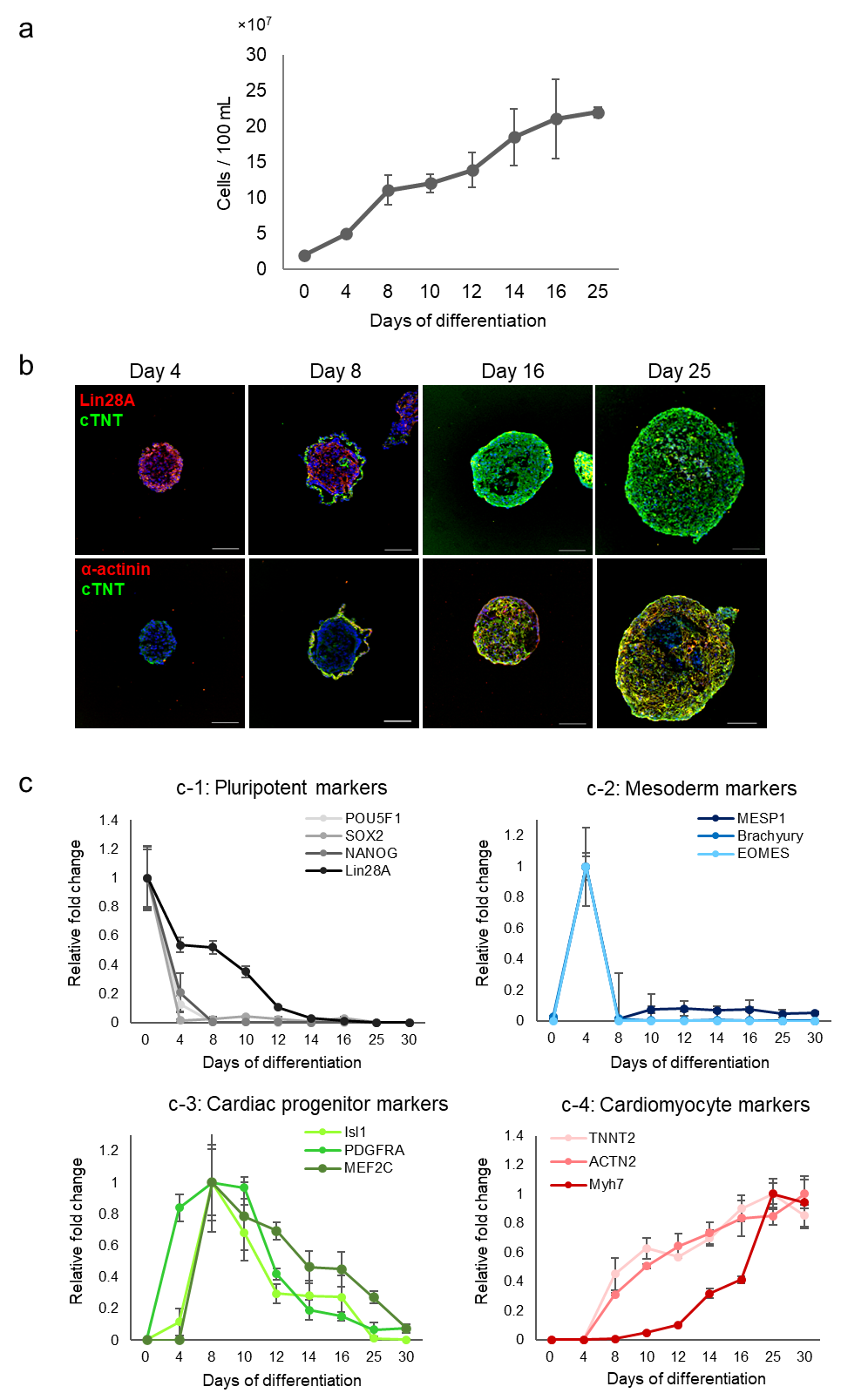
**

**Figure S2. Characterization of hiPSC-CMs**

a: Number of cells per 100 mL bioreactor during cardiomyogenic differentiation induction. The cell numbers were measured 4, 8, 10, 12, 14, 16, and 25 d after induction.

b: Representative immunofluorescence images of differentiated embryoid bodies during cardiomyogenic differentiation induction. Upper panel, staining of Lin28A (red), a marker of undifferentiated stem cells, cardiac troponin T (cTNT; green), a cardiomyocyte marker and nuclei (blue, Hoechst). Lower panel, staining of α-actinin (red) and cTNT (green), both cardiomyocyte markers and nuclei (blue, Hoechst). Scale bars: 20 μm.

c: Gene expression analysis in the context of cardiac differentiation induction.

qPCR was used to evaluate the expression of markers of (c-1) pluripotency (*POU5F1*, *SOX2*, *NANOG,* and *Lin28A*), (c-2) early mesoderm (*MESP1*, *Brachyury*, and *EOMES*), (c-3) cardiac progenitor cells (*Isl1*, *PDGFRA*, and *MEF2C*), and (c-4) cardiomyocytes (*TNNT2*, *ACTN2*, and *MYH7*). Data shown are from one representative experiment for each marker and are normalized to peak expression ± SD.

**
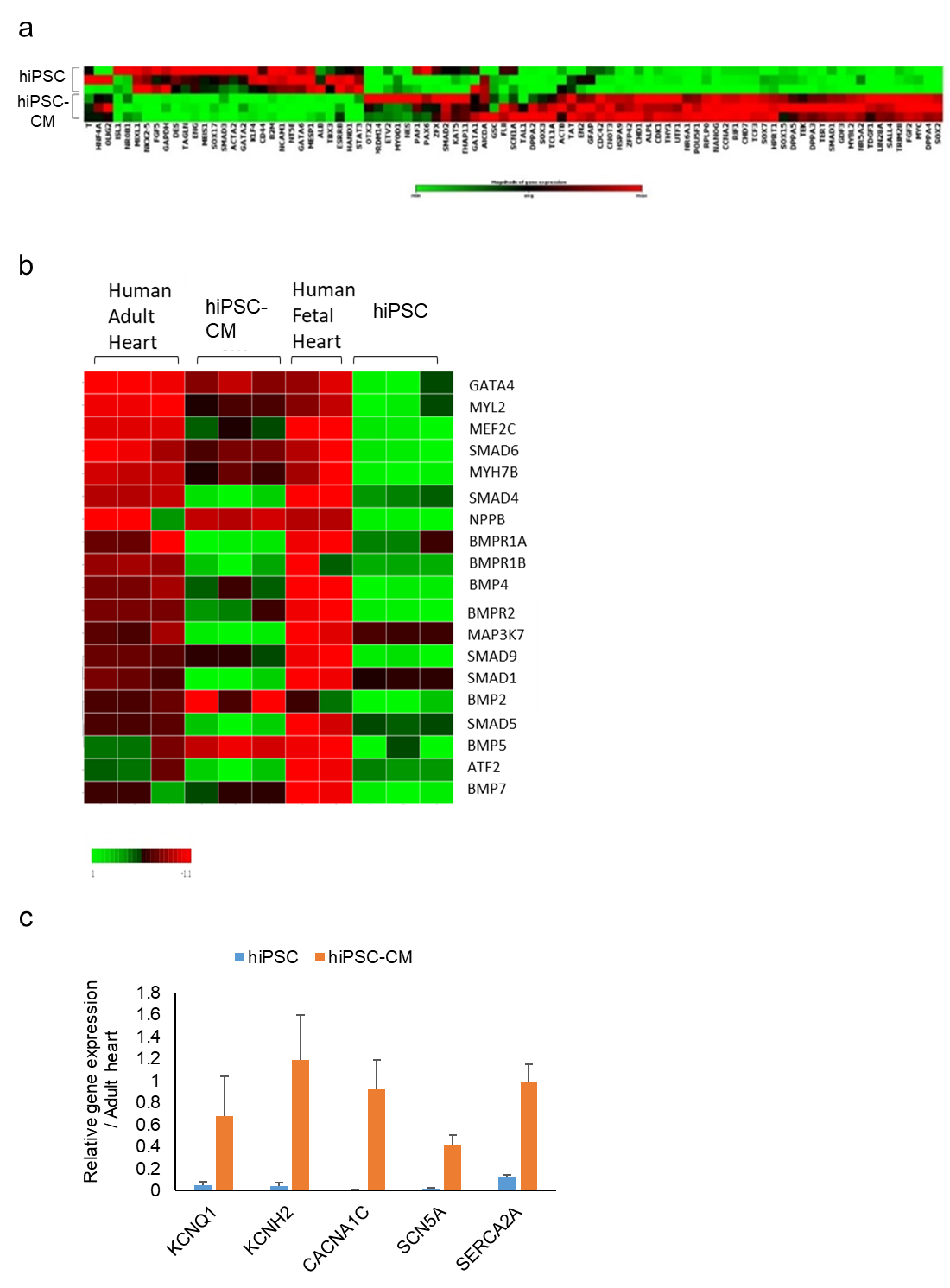
**

**Figure S3. Gene expression pattern of hiPSC-CM**

a: Heatmap of normalized profiling data comparing undifferentiated hiPSCs and hiPSC-CMs. These 84 genes are involved in the stem cell pathway.

b: Heatmap of normalized profiling data comparing undifferentiated hiPSCs, hiPSC-CMs, human fetal heart tissue samples, and human adult heart tissue samples. These 23 genes are cardiac differentiation-associated genes, n = 3; human adult heart, hiPSC-CMs, hiPSC, n = 2; human fetal heart.

c: Relative comparison of the expression of genes associated with ion channels in hiPSCs, hiPSC-CMs, and adult heart tissue samples.

**
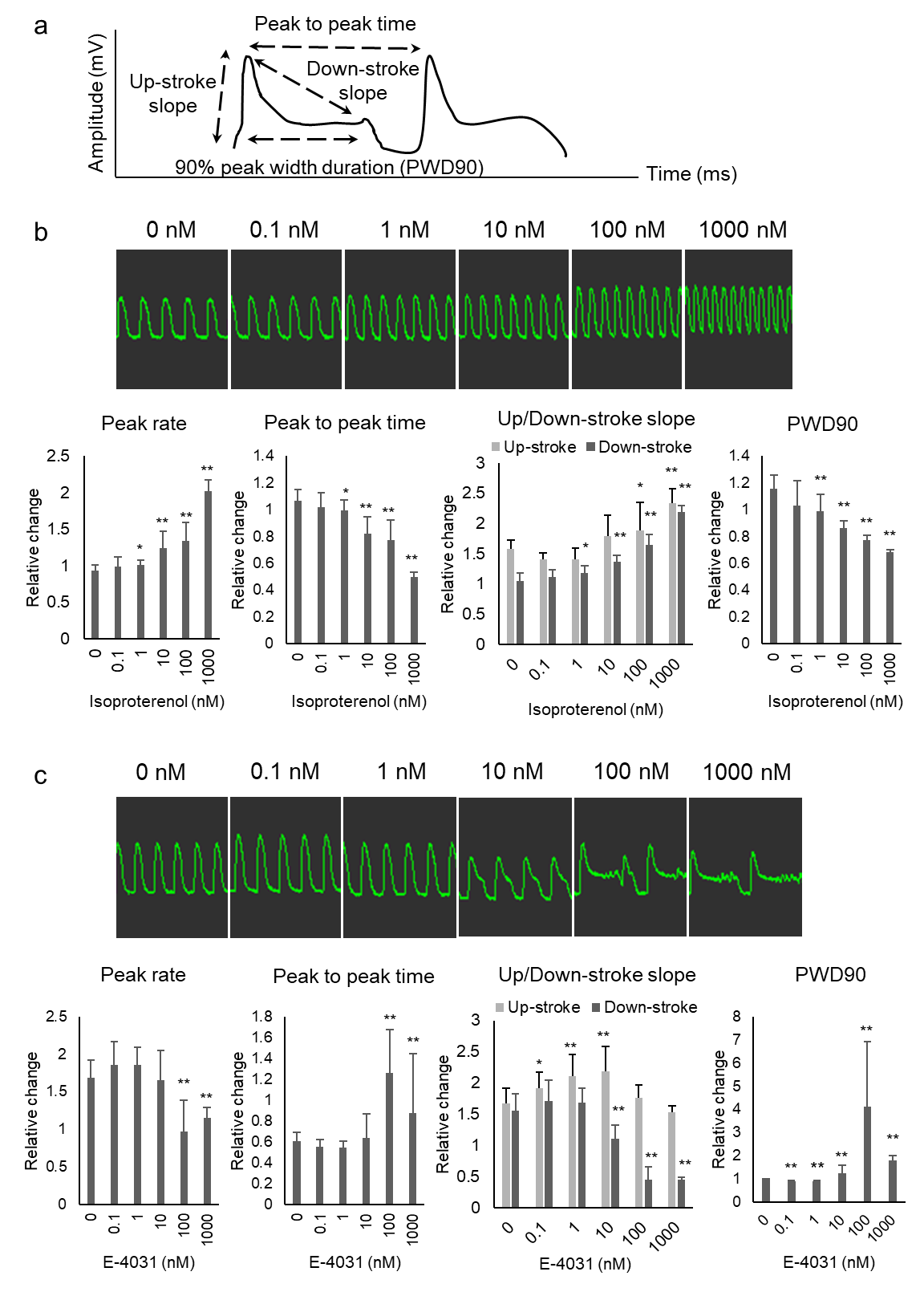
**

**Figure S4. Intracellular calcium levels in hiPSC-CMs following drug administration**

a: Schematic diagram of the calcium transient analysis.

b, c: Representative calcium transient waveform after the addition of isoproterenol (b) or E-4031 (c) and quantitative analysis of the changes in calcium levels after drug administration. The relative change in each parameter, such as the peak rate, peak-to-peak time, upstroke slope, downstroke slope, and 90% peak-width duration (PWD90) after drug administration, was calculated using the predrug treatment samples as the control group. Data are presented as the mean ± SD. **P* < 0.05, ***P* < 0.01 vs. vehicle control group.

**
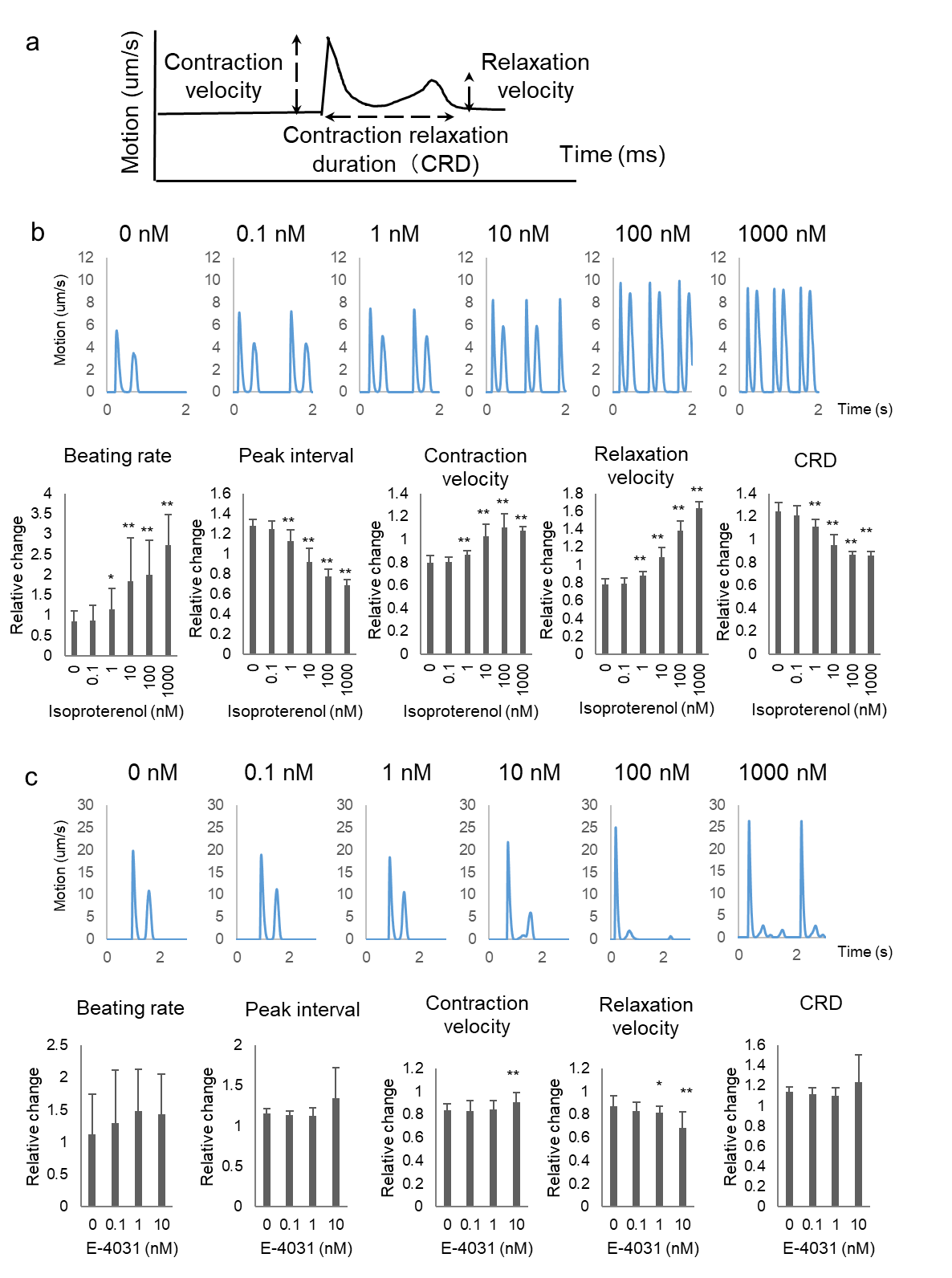
**

**Figure S5. Contraction properties of hiPSC-CMs following drug administration**

a: Schematic diagram of the contraction analysis.

b, c: Representative contraction waveform after the addition of isoproterenol (b) or E-4031 (c) and quantitative analysis of the changes in contraction parameters after drug administration. The relative change in each parameter, such as beating rate, peak interval, contraction/relaxation velocity, and contraction relaxation duration (CRD) after drug administration, was calculated using the predrug treatment samples as the control group. Data are presented as the mean ± SD. **P* < 0.05, ***P* < 0.01 vs. vehicle control.

**
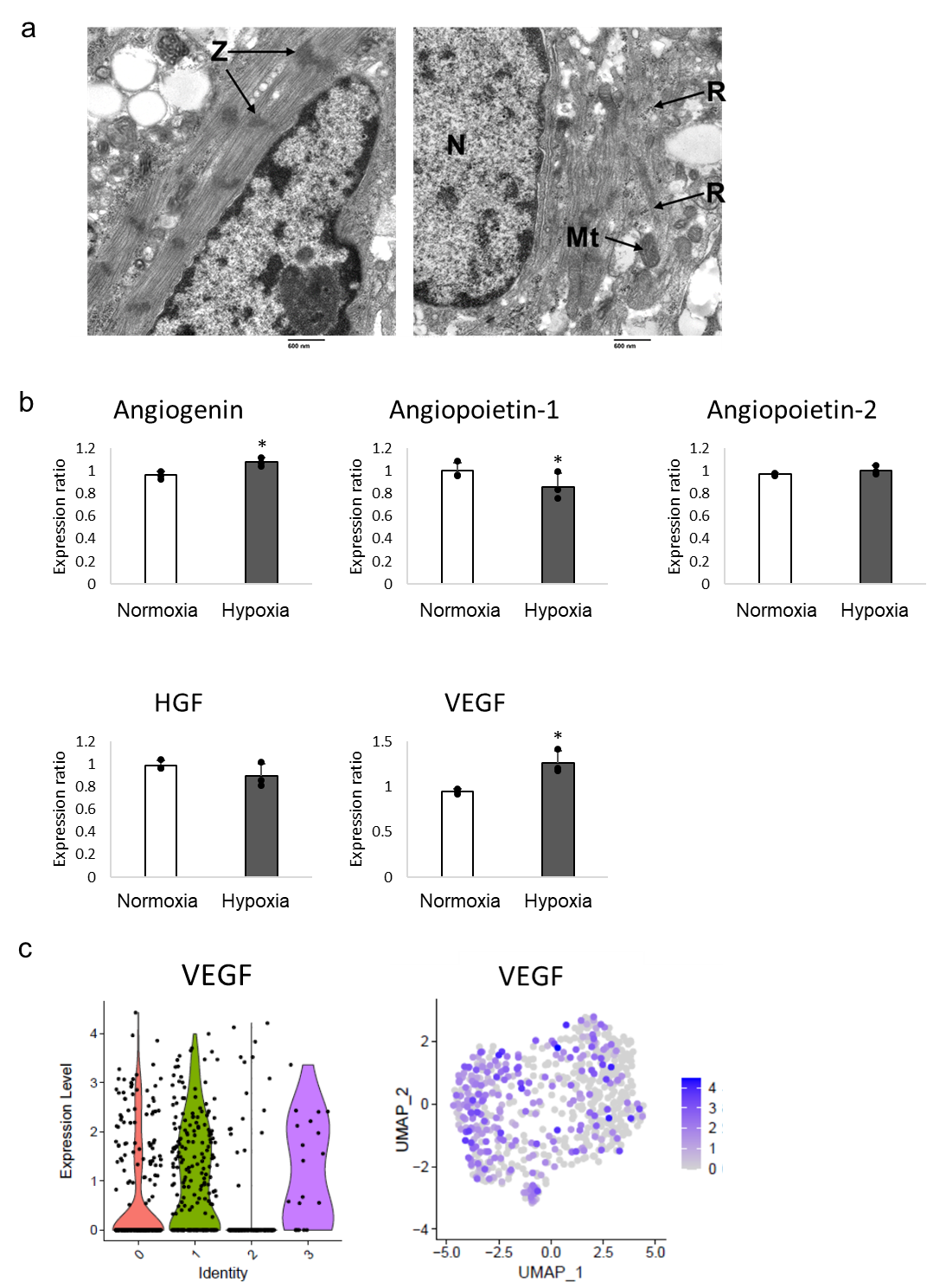
**

**Figure S6. Characterization of the hiPSC-CM patch**

a: TEM images of the hiPSC-CM patch ultrastructural features. Z, Z-line; Mt, mitochondria; R, ribosomes; N, nucleus. Scale bars = 2 µm (magnification: 3,000×).

b: Expression of angiogenic cytokines under normoxic or hypoxic conditions (n = 3).

Data are presented as the mean ± SD. **P* < 0.01.

c: Gene expression of VEGF was analyzed using single-cell RNA-seq.

**
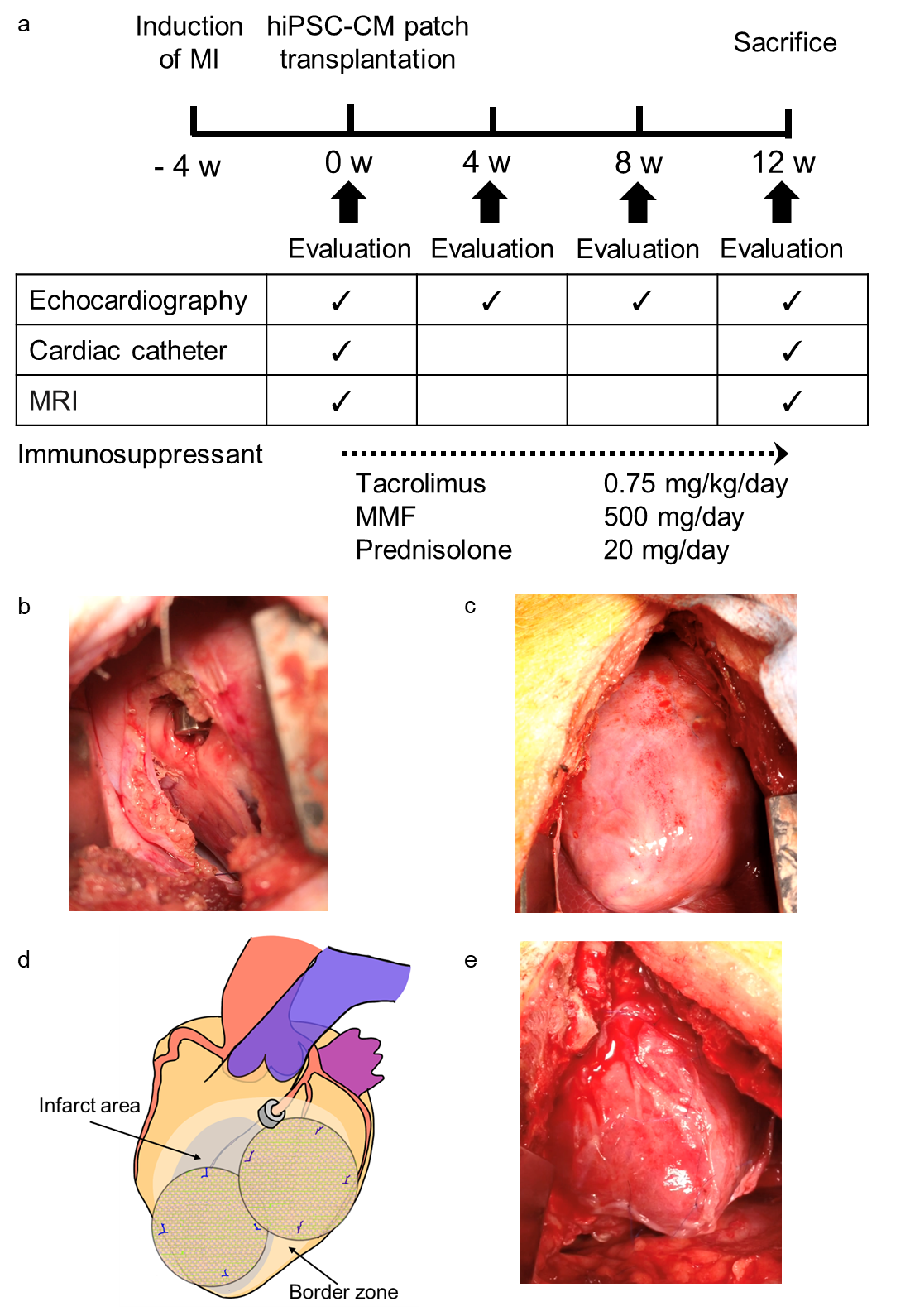
 Figure S7. Experimental protocol for determining efficacy using the porcine MI model**

a: Experimental protocol. hiPSC-CM, human induced pluripotent stem cell-derived cardiomyocyte; MI, myocardial infarction; w, weeks; MMF, mycophenolate mofetil.

b: Representative image of LAD ligation using an ameroid constrictor 4 weeks before implantation. LAD, left anterior descending artery.

c: Representative image of the chronic heart model.

d: Illustration of hiPSC-CM patch implantation.

e: Representative image of hiPSC-CM patch implantation.


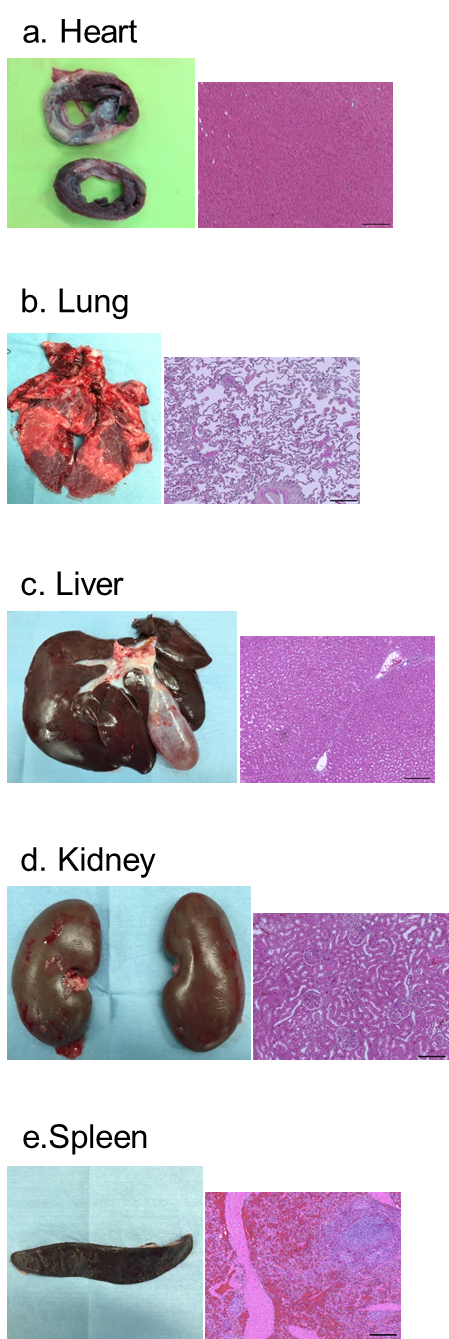


**Figure S8. Posttransplant hiPSC-CM status and effect on organs.**

a: Heart; b: lung; c: liver; d: kidney; e: spleen, and corresponding hematoxylin & eosin-stained images. Scale bars: 200 µm.

**References**

1 Ito E, Miyagawa S, Takeda M, Kawamura A, Harada A, Iseoka H, et al. Tumorigenicity assay essential for facilitating safety studies of hiPSC-derived cardiomyocytes for clinical application. Sci Rep. 2019;9:1881.

2 Ito E, Miyagawa S, Yoshida Y, Sawa Y. Efficient method to dissociate induced pluripotent stem cell-derived cardiomyocyte aggregates into single cells. Methods Mol Biol. 2021;2320:29-33.

3 Sougawa N, Miyagawa S, Fukushima S, Kawamura A, Yokoyama J, Ito E, et al. Immunologic targeting of CD30 eliminates tumourigenic human pluripotent stem cells, allowing safer clinical application of hiPSC-based cell therapy. Sci Rep. 2018;8:3726.

4 Butler A, Hoffman P, Smibert P, Papalexi E, Satija R. Integrating single-cell transcriptomic data across different conditions, technologies, and species. Nat Biotechnol. 2018;36:411-20.

5 Takeda M, Miyagawa S, Fukushima S, Saito A, Ito E, Harada A, et al. Development of in vitro drug-induced cardiotoxicity assay by using three-dimensional cardiac tissues derived from human induced pluripotent stem cells. Tissue Eng Part C Methods. 2018;24:56-67.

6 Teramoto N, Koshino K, Yokoyama I, Miyagawa S, Zeniya T, Hirano Y, et al. Experimental pig model of old myocardial infarction with long survival leading to chronic left ventricular dysfunction and remodeling as evaluated by PET. J Nucl Med. 2011;52:761-8.

7 Kawamura M, Miyagawa S, Fukushima S, Saito A, Miki K, Ito E, et al. Enhanced survival of transplanted human induced pluripotent stem cell-derived cardiomyocytes by the combination of cell sheets with the pedicled omental flap technique in a porcine heart. Circulation. 2013;128(Suppl 1):S87-94.

8 Teichholz LE, Kreulen T, Herman MV, Gorlin R. Problems in echocardiographic volume determinations: Echocardiographic-angiographic correlations in the presence or absence of asynergy. Am J Cardiol. 1976;37:7-11.

9 Fearon WF, Balsam LB, Farouque HM, Caffarelli AD, Robbins RC, Fitzgerald PJ, et al. Novel index for invasively assessing the coronary microcirculation. Circulation. 2003;107:3129-32.

10 Melikian N, Vercauteren S, Fearon WF, Cuisset T, MacCarthy PA, Davidavicius G, et al. Quantitative assessment of coronary microvascular function in patients with and without epicardial atherosclerosis. EuroIntervention. 2010;5:939-45.

11 Obokata M, Nagata Y, Wu VC, Kado Y, Kurabayashi M, Otsuji Y, et al. Direct comparison of cardiac magnetic resonance feature tracking and 2D/3D echocardiography speckle tracking for evaluation of global left ventricular strain. Eur Heart J Cardiovasc Imaging. 2016;17:525-32.

12 Kawamura M, Miyagawa S, Miki K, Saito A, Fukushima S, Higuchi T, et al. Feasibility, safety, and therapeutic efficacy of human induced pluripotent stem cell-derived cardiomyocyte sheets in a porcine ischemic cardiomyopathy model. Circulation. 2012;126:S29-39.

13 Kusakawa S, Machida K, Yasuda S, Takada N, Kuroda T, Sawada R, et al. Characterization of in vivo tumorigenicity tests using severe immunodeficient NOD/Shi-scid IL2Rγnull mice for detection of tumorigenic cellular impurities in human cell-processed therapeutic products. Regen Ther. 2015;1:30-7.

14 Martin M. Cutadapt removes adapter sequences from high-throughput sequencing reads. EMBnet J. 2011;17:10-2.

15 Li H, Durbin R. Fast and accurate short read alignment with Burrows-Wheeler transform. Bioinformatics. 2009;25:1754-60.

16 Yoshida K, Sanada M, Shiraishi Y, Nowak D, Nagata Y, Yamamoto R, et al. Frequent pathway mutations of splicing machinery in myelodysplasia. Nature. 2011;478:64-9.

17 Shiraishi Y, Sato Y, Chiba K, Okuno Y, Nagata Y, Yoshida K, et al. An empirical Bayesian framework for somatic mutation detection from cancer genome sequencing data. Nucleic Acids Res. 2013;41:e89.

18 Wang K, Li M, Hakonarson H. ANNOVAR: Functional annotation of genetic variants from high-throughput sequencing data. Nucleic Acids Res. 2010;38:e164.

19 Sherry ST, Ward MH, Kholodov M, Baker J, Phan L, Smigielski EM, et al. DbSNP: The NCBI database of genetic variation. Nucleic Acids Res. 2001;29:308-11.

20 Exome variant server. NHLBI GO exome sequencing project (ESP), Seattle, WA. <http://evs.gs.washington.edu/EVS/>. Accessed July 2016.

21 1000 Genomes Project Consortium, Auton A, Brooks LD, Durbin RM, Garrison EP, Kang HM, et al. A global reference for human genetic variation. Nature. 2015;526:68-74.

22 Higasa K, Miyake N, Yoshimura J, Okamura K, Niihori T, Saitsu H, et al. Human genetic variation database, a reference database of genetic variations in the Japanese population. J Hum Genet. 2016;61:547-53.

23 Nagasaki M, Yasuda J, Katsuoka F, Nariai N, Kojima K, Kawai Y, et al. Rare variant discovery by deep whole-genome sequencing of 1,070 Japanese individuals. Nat Commun. 2015;6:8018.

24 Stenson PD, Mort M, Ball EV, Evans K, Hayden M, Heywood S, et al. The Human Gene Mutation Database: Towards a comprehensive repository of inherited mutation data for medical research, genetic diagnosis and next-generation sequencing studies. Hum Genet. 2017;136:665-77.

25 Tate JG, Bamford S, Jubb HC, Sondka Z, Beare DM, Bindal N, et al. COSMIC: The catalogue of somatic mutations in cancer. Nucleic Acids Res. 2019;47:D941-7.

26 Sondka Z, Bamford S, Cole CG, Ward SA, Dunham I, Forbes SA. The COSMIC Cancer Gene Census: describing genetic dysfunction across all human cancers. Nat Rev Cancer. 2018;18:696-705.

27 Nakahata T, Okano H. Current perspective on evaluation of tumorigenicity of cellular and tissue-based products derived from induced pluripotent stem cells (iPSCs) and iPSCs as their starting materials (provisional translation). Pharmaceuticals and Medical Devices Agency. 2013. <http://www.pmda.go.jp/files/000152599.pdf>. Accessed 22 Sep 2022.

28 Koboldt DC, Zhang Q, Larson DE, Shen D, McLellan MD, Lin L, et al. VarScan 2: Somatic mutation and copy number alteration discovery in cancer by exome sequencing. Genome Res. 2012;22:568-76.

29 Otsu N. A threshold selection method from gray-level histograms. IEEE Trans Syst Man Cybern. 1979;9:62-6.

30 Rausch T, Zichner T, Schlattl A, Stütz AM, Benes V, Korbel JO. DELLY: Structural variant discovery by integrated paired-end and split-read analysis. Bioinformatics. 2012;28:i333-9.

31 Wang K, Li M, Hadley D, Liu R, Glessner J, Grant SF, et al. PennCNV: An integrated hidden Markov model designed for high-resolution copy number variation detection in whole-genome SNP genotyping data. Genome Res. 2007;17:1665-74.

32 González JR, Rodríguez-Santiago B, Cáceres A, Pique-Regi R, Rothman N, Chanock SJ, et al. A fast and accurate method to detect allelic genomic imbalances underlying mosaic rearrangements using SNP array data. BMC Bioinform. 2011;12:166.

33 Gogarten SM, Bhangale T, Conomos MP, Laurie CA, McHugh CP, Painter I, et al. GWASTools: An R/bioconductor package for quality control and analysis of genome-wide association studies. Bioinformatics. 2012;28:3329-31.

34 Okita K, Yamakawa T, Matsumura Y, Sato Y, Amano N, Watanabe A, et al. An efficient nonviral method to generate integration-free human-induced pluripotent stem cells from cord blood and peripheral blood cells. Stem Cells. 2013;31:458-66.
